# FuNTB: a functional network clustering tool for the analysis of genome-wide genetic variants in *Mycobacterium tuberculosis*

**DOI:** 10.1093/bioinformatics/btaf341

**Published:** 2025-06-11

**Authors:** Axel A Ramos-García, Paulina M Mejía-Ponce, Nelly Sélem-Mojica, Alejandro Santos-Díaz, Emmanuel Martínez-Ledesma, Cuauhtémoc Licona-Cassani

**Affiliations:** Escuela de Ingeniería y Ciencias, Tecnológico de Monterrey, Monterrey, Nuevo León, 64700, México; Centro de Biotecnología FEMSA, Escuela de Ingeniería y Ciencias, Tecnológico de Monterrey, Monterrey, Nuevo León, 64700, México; Centro de Ciencias Matemáticas, Universidad Nacional Autónoma de México, Residencial San José de la Huerta, Morelia, Michoacán, 58089, México; Escuela de Ingeniería y Ciencias, Tecnológico de Monterrey, Monterrey, Nuevo León, 64700, México; Unidad de Biología Integrativa, The Institute for Obesity Research, Tecnológico de Monterrey, Monterrey, Nuevo León, 64700, México; Escuela de Medicina y Ciencias de la Salud, Tecnológico de Monterrey, Monterrey, Nuevo León, 64700, México; Centro de Biotecnología FEMSA, Escuela de Ingeniería y Ciencias, Tecnológico de Monterrey, Monterrey, Nuevo León, 64700, México; Unidad de Biología Integrativa, The Institute for Obesity Research, Tecnológico de Monterrey, Monterrey, Nuevo León, 64700, México; Instituto de Biotecnología, Universidad Nacional Autónoma de México, Cuernavaca, Morelos, 62210, Mexico

## Abstract

**Motivation:**

Tuberculosis (TB), caused by *Mycobacterium tuberculosis* (Mtb), still claims around 1.25 million lives each year. The growing threat of drug resistance—often driven by single‑nucleotide polymorphisms (SNPs) in Mtb genomes underscores the need for high‑quality genomic data and powerful bioinformatics tools. We present FuNTB, a python‑based pipeline that detects non‑synonymous SNPs in Mtb and builds functional network clusters to reveal genotype–phenotype relationships.

**Results:**

FuNTB profiles non‑synonymous SNPs at the gene level across user‑defined phenotypes, pinpointing both shared and unique mutations. It ingests annotated Variant Call Format (VCF) files or MTBseq outputs and merges them with clinical metadata to produce network‑XML files compatible with Cytoscape and Gephi. When applied to the CRyPTIC Mtb collection, FuNTB rapidly recovered established resistance genes and surfaced novel candidates, validating its utility for mapping genotype–phenotype associations.

**Availability and implementation:**

FuNTB is implemented in Python 3.8+ and is freely available under the MIT license at https://doi.org/10.5281/zenodo.15399917.

## 1 Introduction

Advances in sequencing technologies and substantial efforts to understand pathogen transmission have revolutionized the epidemiology of clinically relevant pathogens. These developments have led to extensive genomic databases and bioinformatics tools that correlate genetic variations to phenotypic traits with unprecedented detail (Faksri *et al.* 2016; Rezaei *et al*. 2023). For Mtb, a bacterium that causes (TB), drug resistance is mainly driven by genetic mutations, SNPs and indels, rather than fast-spreading mechanisms like horizontal gene transfer (Nimmo *et al.* 2022). Current genomic catalogues for Mtb have identified specific genetic variants strongly associated with drug-resistance phenotypes (Walker *et al.* 2022). However, a specific tool for analyzing genome-wide mutations and identifying correlations with other phenotypic traits is still missing.

Despite substantial efforts to curb TB, the emergence and spread of drug-resistant *Mycobacterium tuberculosis* (Mtb) strains remain a significant global health challenge (WHO 2020). Accurate, timely diagnosis of drug-resistant TB is especially difficult in high-burden, resource-limited settings, where conventional methods such as culture or phenotypic susceptibility testing are either too slow or prohibitively expensive (Heidary *et al.* 2022). While molecular diagnostics offer high sensitivity and specificity, they are often inaccessible due to technical and cost constraints. Furthermore, interpreting resistance-associated mutations remains a non-trivial task due to the complex genomic background of Mtb. The high genetic heterogeneity of circulating strains and the lack of horizontal gene transfer make it challenging to distinguish causative mutations from neutral variation, particularly for second-line drugs. These challenges demand analytical approaches that can pinpoint biologically relevant resistance mechanisms while filtering out background noise (Rezaei *et al*. 2023; Villar-Hernández *et al.* 2023).

The concept of a functional network has greatly advanced system studies, establishing that relationships between elements can influence the dynamics of a phenomenon. In the past, network approaches have driven integrative omics analyses (Nelson *et al.* 2021). In this context, FuNTB is designed to analyze non-synonymous variations in Mtb strains which primary goal is to construct functional networks highlighting genes with non-synonymous mutations present or absent between groups of samples with contrasting phenotypes. Built on the output from MTBSeq (Nelson *et al.* 2021), a widely used tool for detecting whole genome variants, FuNTB finds both unique and shared genetic mutations, aiding in identifying molecular distinctions tied to diverse phenotypic conditions.

FuNTB visualizes non-synonymous mutations in Mycobacterium tuberculosis by mapping genes and phenotype-defined sample groups as network nodes whose sizes reflect the Combined Alteration Impact Score (CAIS) and whose colors indicate phenotype, thereby highlighting genetic relationships across contrasting groups. This integrated framework enables researchers to compare shared and unique mutation signatures systematically and to generate hypotheses about gene–phenotype associations and underlying biological mechanisms. By streamlining the comparative analysis of mutation patterns in Mtb, FuNTB enhances our understanding of how specific genetic alterations drive phenotypic diversity, supporting targeted investigations in infectious-disease research.

## 2 Software implementation

FuNTB is a standalone Python tool that converts MTBseq v1.1.0 outputs or annotated VCFs plus clinical metadata into XML‐based network graphs with minimal coding required. It consists of three scripts: (i) a variation-dictionary generator that parses SNPs into a Python dict mapping each sample ID to its genes and mutation positions with frequencies; (ii) a clinical-grouping script that filters samples by user-selected phenotypic features and exports group-specific ID lists; and (iii) a network-construction script that integrates these outputs to build and visualize gene–phenotype association network.

**Figure 1.**
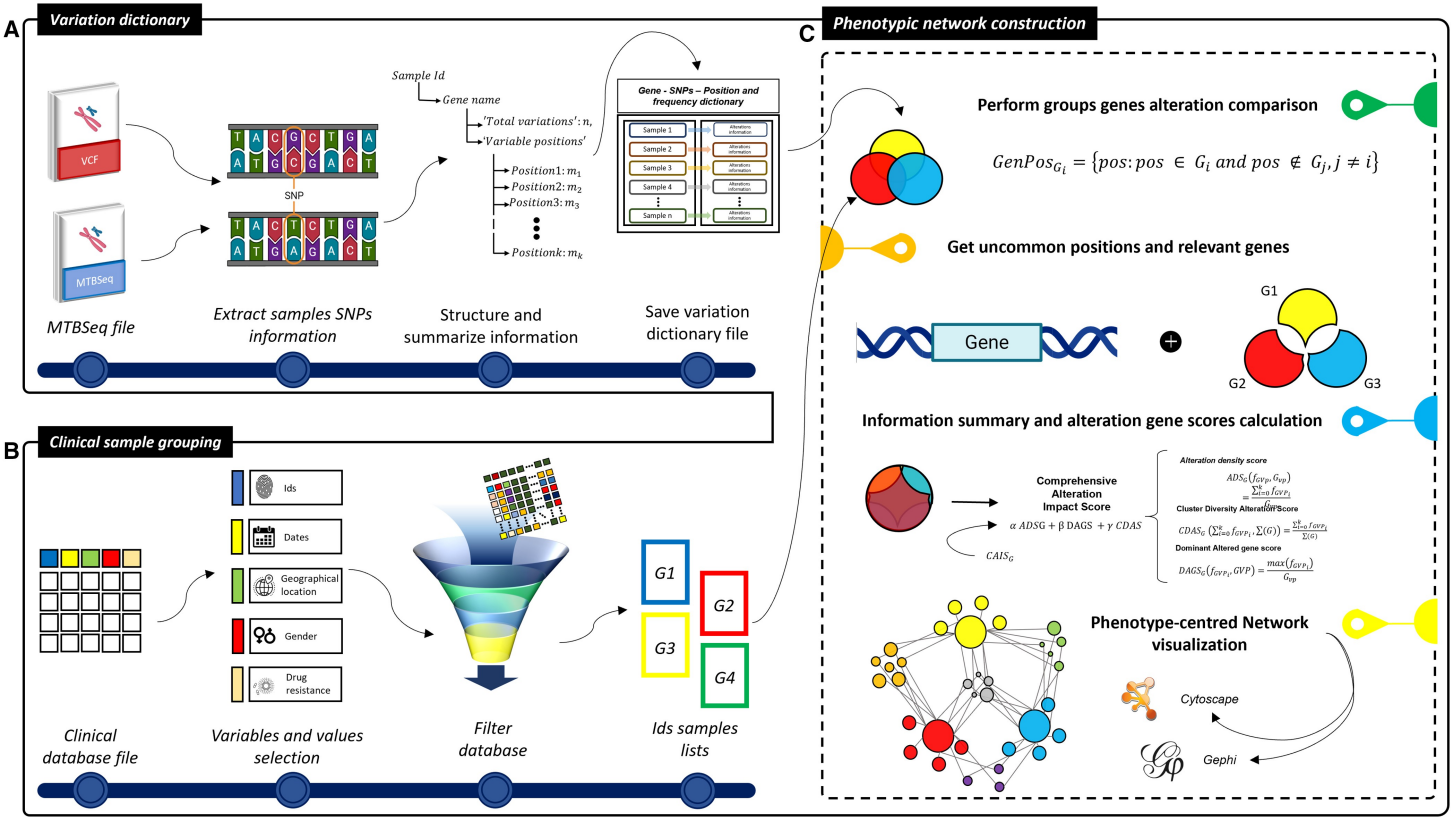
FuNTB’s workflow comprises three key scripts: (A) the Variation Dictionary script parses MTBseq outputs or annotated VCFs to build a Python object that records, for each gene, all non-synonymous SNP positions and their frequencies; (B) the Clinical Sample Grouping script reads sample metadata and, based on user-defined clinical or experimental criteria, iteratively filters and exports text files listing sample IDs for each phenotype group; and (C) the Phenotypic Network Construction script combines the variation dictionary with these group lists and Pareto-front parameters to perform pairwise exclusion of shared variant positions, retain genes bearing unique alterations per phenotype, compute prioritization metrics (ADS, DAGS, CDAS, and CAIS), and output phenotype-centered networks in GML, GraphML, and GEXF formats for visualization and downstream analysis.

The FuNTB workflow Fig. 1 begins by parsing MTBseq outputs to extract and filter non-synonymous SNPs, organizing them by sample in dictionaries. Samples are then grouped by clinical variables to create phenotype-specific gene lists, from which unique mutated positions are identified. Next, phenotype-centered networks are built: genes and phenotype groups become nodes sized by mutation-frequency ratios and color-coded by group. Genes are ranked via Pareto‐set criteria, retaining only top candidates, and the final networks are exported in GML, GraphML, and GEXF formats.

### 2.1 Input and data preparation

#### 2.1.1 Data requirements

To execute “FuNTB,” two major inputs need to be supplied. The first is either an MTBseq v.1.1.0 Output File or a list/series of VCF files that hold sample IDs and SNPs information, including nucleotide change and, where applicable, non-synonym amino acid change. Second, the other is a clinical data table including sample-specific information that could range from gender, extraction date, comorbidities, or drug susceptibility, among others. The clinical data need to be standardized to allow compatibility and proper processing, including standardized formatting and encoding across entries (with further explanation in Supplementary Note).

#### 2.1.2 Data processing

The SNP information from MTBseq or VCF files is processed by the algorithm, from which gene-specific alteration information is extracted and formatted into a Python dictionary. Each gene is entered as a key, while non-synonymous SNP information (e.g. original amino acid, gene position, amino acid change) and mutation frequency are documented for each position of uniqueness. To generate sample lists, user assistance is solicited for defining the number of groups and choosing one value for each clinical variable. The dataset is progressively filtered according to these choices, and the sample lists generated in this manner are output as distinct text files, one for each group.

#### 2.1.3 Gene comparison framework

To The algorithm first builds separate data frames for each phenotypic group, summarizing altered genes, mutation sites, and their frequencies from the variation dictionary. It then performs pairwise group comparisons to filter out shared mutations, retaining only genes with unique variant positions. These genes become nodes in a phenotype-centered network—linked to their respective group hubs—with node size, color, and edge attributes encoding four prioritization metrics: Alteration Density Score (ADS), Dominant Altered Gene Score (DAGS), Cluster Diversity Alteration Score (CDAS), and the composite Comprehensive Alteration Impact Score (CAIS). This streamlined workflow highlights phenotype-specific genetic signatures and ranks genes by their potential relevance to observed phenotypic differences.

#### 2.1.4 Network construction

FuNTB constructs a phenotype-centered network with two node types: gene nodes represent genes harboring phenotype-specific non-synonymous mutations and are sized by the Comprehensive Alteration Impact Score (CAIS), a weighted composite of three metrics—Alteration Density Score (ADS; total variant frequency divided by the number of mutated positions, where low ADS indicates hotspot clustering in functionally critical regions and high ADS reflects dispersed, likely background, variation), Dominant Altered Gene Score (DAGS; total variant frequency normalized by the number of altered genes within the same phenotypic cluster, with high DAGS highlighting disproportionately prevalent variants likely driving phenotype), and Cluster-Diversity Alteration Score (CDAS; total variant frequency divided by the number of phenotypic clusters with any mutation, where low CDAS signifies phenotype specific alterations and high CDAS suggests broad adaptation or non-specific background variation). Detailed equations are provided in the Supplementary Notes. Larger gene nodes (e.g. rpoB in rifampicin resistance) therefore indicate genes with concentrated, prevalent, and phenotype-specific alterations, while smaller nodes reflect more ubiquitous or background mutations. Phenotype ego-nodes serve as fixed size hubs (e.g. “Drug-Resistant” or “Susceptible”), colored by phenotypic group (red for resistant, blue for susceptible). This dual-node architecture (Fig. 5, available as supplementary data at *Bioinformatics* online) enables intuitive visualization of mutation–phenotype relationships by highlighting high-CAIS genes as potential drivers of phenotypic differences.

#### 2.1.5 Pareto optimization and coefficients calibration

FuNTB prioritizes genes via multi-objective Pareto optimization across four metrics—ADS (minimized), DAGS, CDAS, and CAIS (maximized)—with metric weights calibrated using Grid, Randomized, and Bayesian searches on CRyPTIC data for isoniazid- and rifampicin-resistant samples (see Supplementary Notes). We tuned the CAIS coefficients to concentrate known resistance genes in the top Pareto fronts, using a fitness score that rewards target genes appearing within the first five fronts. Bayesian optimization yielded the best result (fitness = 0.45), placing nine targets in the first front and three in the second. Detailed equations and full optimization outcomes are provided in the Supplementary Notes.

#### 2.1.6 Output and interpretation

FuNTB generates results in multiple network formats (GML, GRAPHML, GEXF) to allow easy access and processing in graph-specific tools for visualization. Once the network is processed, nodes can be displayed with different colors and sizes, representing their connections to one or more phenotypic nodes. If a gene node is connected to only one phenotypic node, it suggests that the gene does not show unique alterations in other ego-nodes. This could indicate that the gene is a potential candidate signature for the associated phenotype. On the other hand, nodes connected to multiple phenotypic nodes represent genes with alterations shared across different phenotypic groups, which may provide insights into shared genetic traits or broader biological roles.

#### 2.1.7 Use case

To demonstrate the capabilities of FuNTB, we applied FuNTB on CRyPTIC (Yousef 2020) dataset, extracting Isoniazid, Rifampicin and Sensitive samples sets from the 1436 Mtb isolates processed with MTBseq. The goal was to explore the genetic variations between these different phenotypic groups to generate a network highlighting those genes with SNPs previously associated with providing antibiotic resistance.

#### 2.1.8 Data preprocessing

From the complete dataset, we defined three phenotypic groups by drug-resistance profile: Group A consists of 645 isolates exhibiting monoresistance to isoniazid (INH), Group B comprises 144 isolates with monoresistance to rifampicin (RIF), and Group C includes 647 isolates sensitive to both drugs (SEN). MTBseq v.1.1.0 (Kohl *et al.* 2018) allowed us to map the position of SNPs to specific genes in each isolate.

#### 2.1.9 Biological insights

Our analysis identified several key resistance-associated genes within the top-ranked candidates, including *rpoA* (Khan *et al.* 2021), *pncA* (Ghosh *et al.* 2020), *katG* (Escuyer *et al.* 2001), *rpoB* (Hameed *et al.* 2022), *fgd1* (Nimmo *et al.* 2022), *embA* (Escuyer *et al.* 2001), *embB* (Escuyer *et al.* 2001), *rpoC* (Khan *et al.* 2021), and *rpsL* (Ghosh *et al.* 2020) in the first Pareto Front, as well as *gyrB* (Nguyen *et al.* 2023), embC (Escuyer *et al.* 2001), and ethA (Nimmo *et al.* 2022) in the second part. These genes have established roles in resistance to first and second line antituberculosis drugs, such as Isoniazid and Rifampicin, Pyrazinamide (Carter *et al.* 2024), Delamid/Pretomanid (Nguyen *et al.* 2023) and Ofloxacin (Sun *et al.* 2014). Notably, FuNTB also prioritized previously unexplored genes, suggesting their potential involvement in novel resistance mechanisms. These findings provide new avenues for investigating Mtb pathogenesis and drug resistance mechanisms.

#### 2.1.10 Network visualization

FuNTB exported the phenotypic-centered network in GML, GRAPHML, and GEXF formats, to subsequently be visualized in Cytoscape (Otasek *et al.* 2019) or Gephi. The resulting network provided a clear graphical representation of the genetic alterations across the three phenotypic groups, with node size reflecting CAIS values and node color indicating the phenotypic group association.

## 3 Conclusion

FuNTB is an open-source framework for analyzing non synonymous variations in *Mycobacterium tuberculosis*, integrating MTBseq genomic data and user-defined clinical parameters to build phenotype-centered networks and pinpoint candidate genes via metrics like CAIS. Applied to a CRyPTIC Mtb subset, it revealed both shared and phenotype-specific genetic signatures and produced biologically meaningful networks. Although validated for Mtb, its modular design supports extension to other organisms.

Future work will add synonymous and structural variants, link resistance databases, and automate annotation to better prioritize functionally relevant mutations. We will also incorporate machine-learning modules for refined gene ranking and enhanced phenotype prediction, broadening FuNTB’s utility in clinical microbiology and precision infectious-disease research.

## Supplementary Material

btaf341_Supplementary_Data

## Data Availability

FuNTB is implemented in Python 3.8+ and is freely available under the MIT license at https://doi.org/10.5281/zenodo.15399917.

## References

[btaf341-B1] Bastian S , Heymann M, Jacomy. Gephi: an open source software for exploring and manipulating networks. In: *International AAAI Conference on Weblogs and Social Media*, San José, California, USA. 2009.

[btaf341-B2] Brimacombe M , Hazbon M, Motiwala AS et al Antibiotic resistance and single-nucleotide polymorphism cluster grouping type in a multinational sample of resistant mycobacterium tuberculosis isolates. Antimicrob Agents Chemother 2007;51:4157–9. 17846140 10.1128/AAC.00619-07 PMC2151444

[btaf341-B3] Carter JJ , Walker TM, Walker AS et al Prediction of pyrazinamide resistance in mycobacterium tuberculosis using structure-based machine-learning approaches. JAC Antimicrob Resistance 2024;6:dlae037. 10.1093/jacamr/dlae037 PMC10946228 38500518

[btaf341-B4] Escuyer MA , Lety JB, Torrelles KH et al The role of the emba and embb gene products in the biosynthesis of the terminal hexaarabinofuranosyl motif of mycobacterium smegmatis arabinogalactan. J Biol Chem 2001;276:48854–62. 11677227 10.1074/jbc.M102272200

[btaf341-B5] Faksri JH , Tan A, Chaiprasert YY et al Bioinformatics tools and databases for whole genome sequence analysis of mycobacterium tuberculosis. infection, genetics and evolution. J Mol Epidemiol Evol Genet Infect Dis 2016;45:359–68. 10.1016/j.meegid.2016.09.013 27637931

[btaf341-B6] Ghosh A , N S, Saha S. Survey of drug resistance associated gene mutations in mycobacterium tuberculosis, eskape and other bacterial species. Sci Rep 2020;10:8957. 32488120 10.1038/s41598-020-65766-8 PMC7265455

[btaf341-B7] Hameed C , Fang Z, Liu Y et al Characterization of genetic variants associated with rifampicin resistance level in mycobacterium tuberculosis clinical isolates collected in guangzhou chest hospital, China. Infect Drug Resist 2022;15:5655–66. 36193294 10.2147/IDR.S375869 PMC9526423

[btaf341-B8] Heidary M , Shirani M, Moradi M et al Tuberculosis challenges: resistance, co-infection, diagnosis, and treatment. Eur J Microbiol Immunol (Bp) 2022;12:1. 35420996 10.1556/1886.2021.00021 PMC9036649

[btaf341-B9] Khan JE , Phelan MT, Khan S et al Characterization of rifampicin-resistant mycobacterium tuberculosis in khyber pakhtunkhwa, Pakistan. Sci Rep 2021;11:15100. 34282262 10.1038/s41598-021-94687-3 PMC8290046

[btaf341-B10] Kohl C , Utpatel V, Schleusener MR et al Mtbseq: a comprehensive pipeline for whole genome sequence analysis of mycobacterium tuberculosis complex isolates. PeerJ 2018;6:e5895. 30479891 10.7717/peerj.5895 PMC6238766

[btaf341-B11] Nelson S , Talarico S, Poonja CJ et al Mutation of mycobacterium tuberculosis and implications for using whole-genome sequencing for investigating recent tuberculosis transmission. Front Public Health 2021;9:790544. 35096744 10.3389/fpubh.2021.790544 PMC8793027

[btaf341-B12] Nguyen QH , Nguyen TNT, Nguyen RM et al Pretomanid resistance: an update on emergence, mechanisms and relevance for clinical practice. Int J Antimicrob Agents 2023;62:106953. 37595848 10.1016/j.ijantimicag.2023.106953

[btaf341-B13] Nimmo J , Millard V, Faulkner J et al Evolution of mycobacterium tuberculosis drug resistance in the genomic era. Front Cell Infect Microbiol 2022;12:954074. 36275027 10.3389/fcimb.2022.954074 PMC9585206

[btaf341-B14] Otasek D , Morris JH, Bouças J et al Cytoscape automation: empowering workflow-based network analysis. Genome Biol 2019;20:185. 31477170 10.1186/s13059-019-1758-4 PMC6717989

[btaf341-B15] Sun Y , Xu Y, Sun Y et al Ofloxacin resistance in mycobacterium tuberculosis is associated with efflux pump activity independent of resistance pattern and genotype. Microb Drug Resist 2014;20:525–32. 24940805 10.1089/mdr.2013.0171

[btaf341-B1500] Rezaei N, Hosseini N-S, Saghazadeh A et al Tuberculosis: Integrated Studies for a Complex Disease 2050. In: Rezaei N (ed.), *Tuberculosis. Integrated Science*, Vol 11. Cham: Springer, 2023.

[btaf341-B16] Villar-Hernández A , Ghodousi O, Konstantynovska R et al Tuberculosis: current challenges and beyond. Breathe (Sheff) 2023;19:220166. 37334103 10.1183/20734735.0166-2022 PMC10270564

[btaf341-B17] Walker P , Miotto CU, K¨oser PW, Seq&Treat Consortium et al The 2021 who catalogue of mycobacterium tuberculosis complex mutations associated with drug resistance: a genotypic analysis. The Lancet. Microbe 2022;3:e265–e273. 35373160 10.1016/S2666-5247(21)00301-3 PMC7612554

[btaf341-B18] WHO. Tuberculosis, 2020 (29 October 2024, date last accessed).

[btaf341-B19] Yousef. Cryptic disseminated tuberculosis: a secondary analysis of a previous hospital-based study. Tanaffos 2020;19:45–9. 33101431 PMC7569501

