# Supplementary material for: FuNTB: a functional network clustering tool for the analysis of genome-wide genetic variants in Mycobacterium tuberculosis
Source: Bioinformatics. 2025 Jun 11;41(7):btaf341. doi: 10.1093/bioinformatics/btaf341 (PMC12255883; doi:10.1093/bioinformatics/btaf341)
Supplement: btaf341_Supplementary_Data [file btaf341_supplementary_data.zip › FunTB_supplementary_notes.docx]

**FuN-TB: A computational tool for network clustering of *Mycobacterium tuberculosis* gene variation in contrasting phenotype groups**

Axel A. Ramos-García^1^, Paulina M. Mejía-Ponce^1,*^, Nelly Selem Mojica^2^, J. Alejandro Santos-Díaz^1^, Emmanuel Martínez-Ledesma^3,4^ and Cuauhtémoc Licona-Cassani^1,3,5^**^ş^**

^1^ Escuela de Ingeniería y Ciencias, Tecnológico de Monterrey, N.L. México.

^2^ Centro de Ciencias Matemáticas, UNAM, México

^3^ Tecnologico de Monterrey, Institute for Obesity Research., N.L. México.

^4^ Escuela de Medicina y Ciencias de la Salud, Tecnológico de Monterrey, Monterrey, N.L. México.

^5^Instituto de Biotecnología, Universidad Nacional Autónoma de México, Cuernavaca, Morelos, Mexico

*Current address: Laboratory for Pathogenesis of Clinical Drug Resistance and Persistence, San Diego State University, San Diego, CA, USA

**^ş^Correspondence:**

Cuauhtémoc Licona-Cassani. Centro de Biotecnología FEMSA, Escuela de Ingeniería y Ciencias, Tecnológico de Monterrey, N.L. México. [clicona@tec.mx](mailto:clicona@tec.mx)

**Supplementary Notes**

**Table of Contents**

**Note 1 – Introduction.**

**Note 2 – Pipeline Workflow.**

- **Step 1:** Variation Dictionary Creation.
- **Step 2:** Sample List Creation.
- **Step 3:** Phenotype-Centric Network Construction.

**Note 3 –** **Algorithmic Implementation.**

- **Algorithm 1:** Variation Dictionary Creation.
- **Algorithm 2:** Sample List Creation.
- **Algorithm 3:** Network Construction.

**Note 4 –** **Metrics’ Equations and Interpretation.**

**Note 5 – Statistical** **validation.**

**Note 6 – Pareto front strategy to identify outstanding genes.**

**Note 7 – Network Visualization.**

**Note 1 – Introduction.**

These supplementary notes provide comprehensive details about the methodologies, extended results, and algorithmic approaches presented in the main manuscript. The primary goal of this document is to enhance the reproducibility and transparency of all experiments conducted, while offering a more in-depth understanding of FunTB's design, operation, and applications. By including extended data, code implementations, and additional analyses, we aim to facilitate the adoption of FunTB by the research community and clarify the nuances of its functionality.

**Note 2 – Pipeline Workflow.**

1. **Pipeline overview**

The FunTB pipeline is comprised of three scripts focused on specific stages: (i) Variation dictionary creation, (ii) Sample lists creation, and (iii) Phenotype centred network.

**
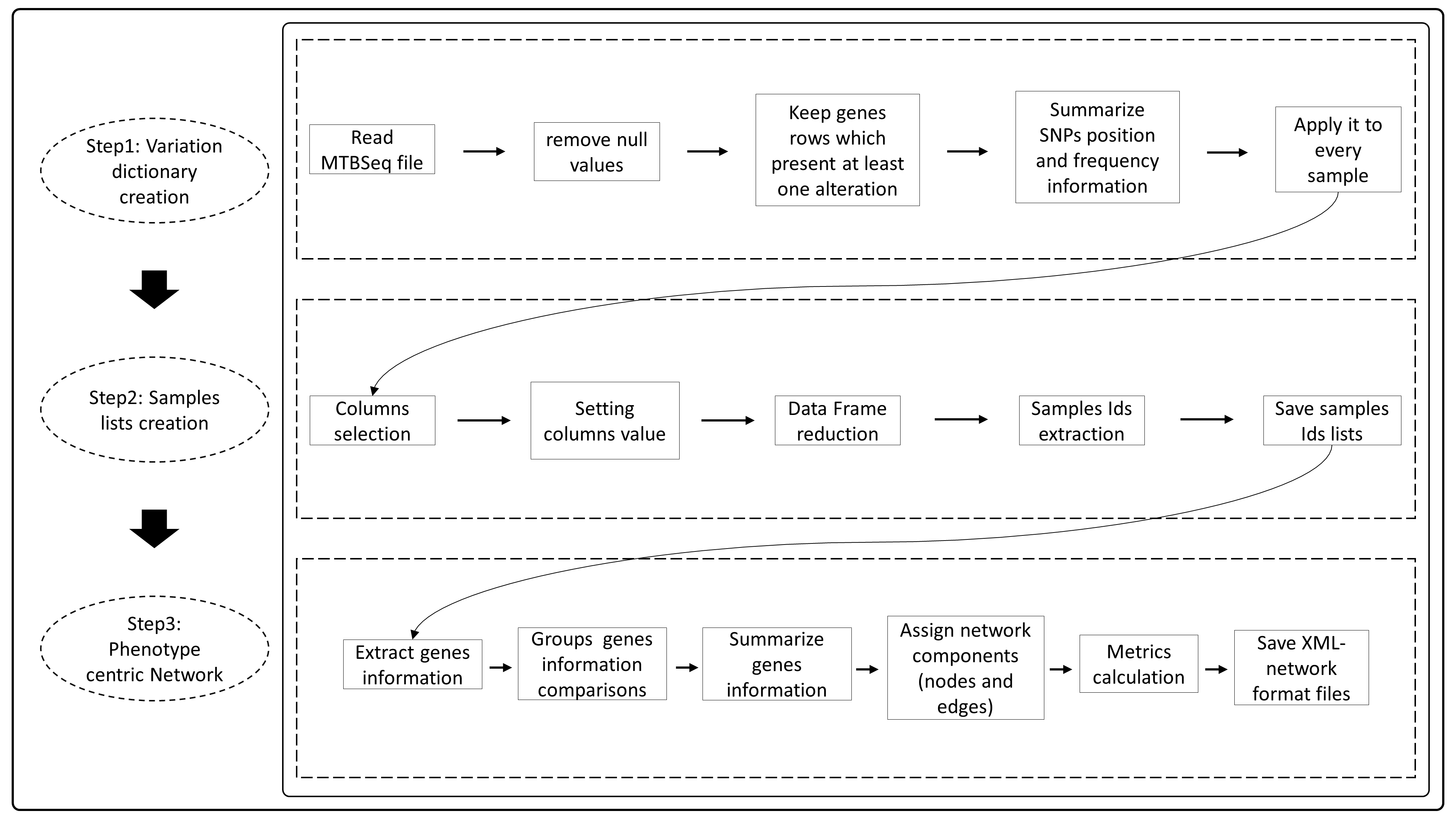
**

Supplementary Figure 1 High-level flow diagram of FunTB pipeline **Step 1:** Variation dictionary creation – The pipeline processes MTBseq SNPs data by removing null values and scanning each gene for the presence of non-synonymous alterations. Findings are stored in a Python dictionary, recording the altered positions and their corresponding frequencies. **Step 2: Samples lists creation:** This script reads a clinical data table and iterates through each column to filter sample IDs based on specified variable values. The result is a list of samples matching the selected criteria. **Step 3: Phenotype centric network:** Using files from previous steps, the genomic information for each sample group is extracted and summarized into a dataframe, listing genes and alteration frequencies. A pairwise comparison is performed to exclude common altered positions, retaining only unique ones. These unique alterations are converted into nodes, linked to phenotype-specific ego nodes, and assigned features such as size. The CAIS metric is calculated to define node size, providing a visual representation of network characteristics.

**FunTB - Variation dictionary creation script:**

**Objective:** To generate a dictionary object summarizing genes and their alteration frequencies from a MTBSeq output file.

**inputs:**

-- MTBSeq_file | MTBSeq output file with variants information.

-- Clinical_data_file | Relational data table that contains clinical information and sample conditions.

-- Dictionary_file_name | Output file name (string).

**FunTB - Sample lists creation inputs:**

**Objective:** To create a series of sample ID lists, each saved as a separate text file, representing groups with identical phenotypic features. These lists enable precise selection and categorization of samples based on shared characteristics.

-- Clinical_data_file | Relational data table that contains clinical information and sample conditions.

-- Selected_columns | Variables of interest to perform the filter sample selection (list of strings).

**FunTB – Phenotype centred networks inputs:**

**Objective:** To produce a series of XML-format files that encode the relational network, illustrating the associations between genes and phenotypes. These files facilitate the visualization and analysis of complex relationships, enhancing the interpretability of the data.

-- Network_name | Output network format files name (string).

-- Variation_dictonary_file | Plain text file containing summarized samples variation information (txt).

-- Pareto_fronts | Number of Pareto fronts of genes included within final network structure (int).

-- alpha | Alteration density score ponderation factor (float).

-- beta | Cluster Diversity Alteration Score ponderation factor (float).

-- gamma | Dominant Altered gene score ponderation factor (float).

-- group_lists | Groups samples Id’s list files names (strings).

1. **Step1: Variation dictionary creation.**

In this processing step, FunTB aims to generate a dictionary object from an MTBSeq output or annotated VCF files. It extracts sample IDs and retains genes with non-synonymous alterations for each sample. The mutations are represented using an acronym format: the original amino acid abbreviation, followed by the chromosomal position of the alteration, and concluding with the resulting amino acid abbreviation. This acronym is then utilized to isolate the relevant position and compute the final frequency of alterations per gene for each sample. The process of this script is illustrated in Supplementary Figure 2.


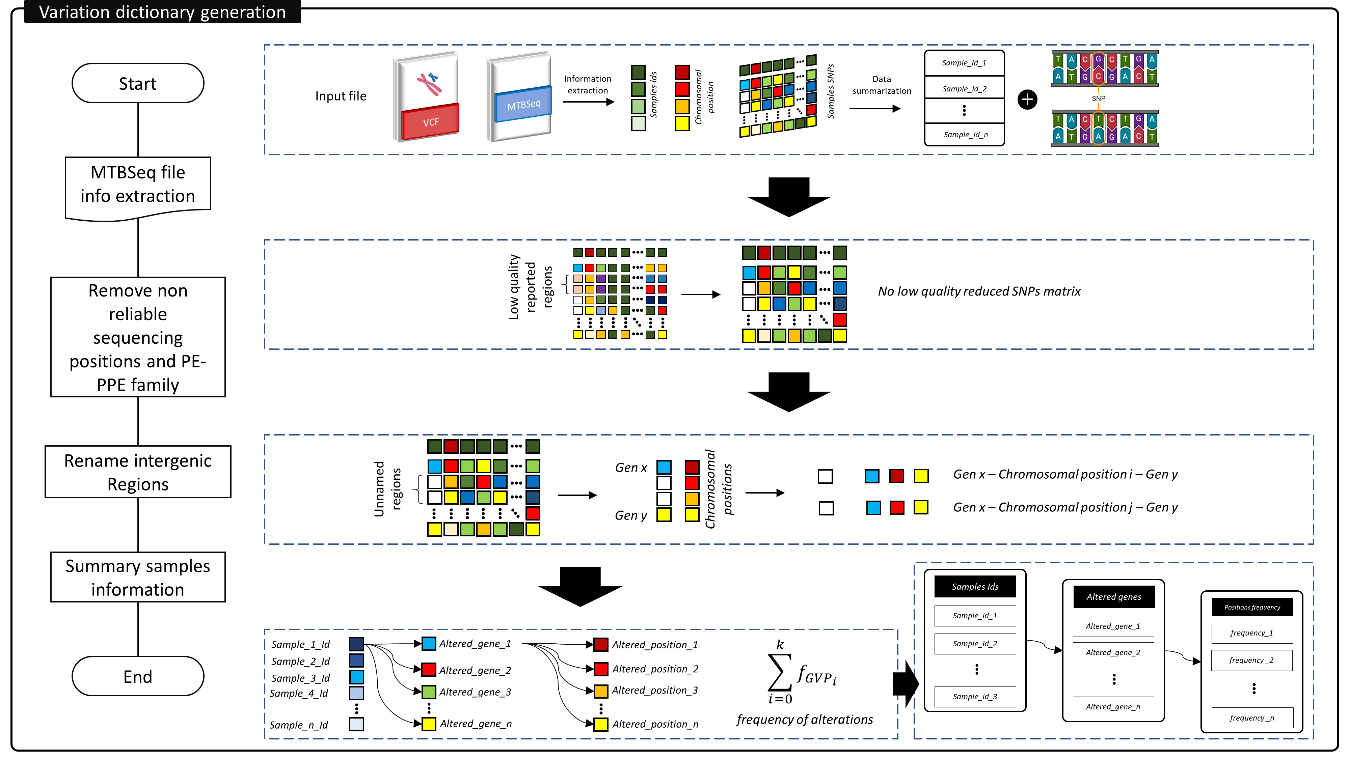


*Supplementary Figure 2 illustrates the initial script of FunTB, showcasing a flowchart on the right and a schematic representation of each stage on the left. The process begins by extracting relevant data from the MTBSeq output file and proceeds to filter out positions linked to PE and PPE gene families. Next, empty cells in the sample ID column are replaced with appropriate names, followed by concatenating the gene name, position, and the subsequent gene name for clarity. Finally, the script compiles all sample alteration details into a dictionary object, where the sample ID serves as the primary key. Within this structure, genes act as the first-level nested keys, with the positions of gene mutations and their corresponding frequencies meticulously recorded.*

1. **Step2: Samples list creation.**

In this processing step FunTB looks to generate a series of plain text files which have the samples Ids that correspond to selected clinical variables values, the user needs to provide the number of the desired groups to create, the names of the groups and as an optional parameter the name of the columns of the clinical variables of interest.


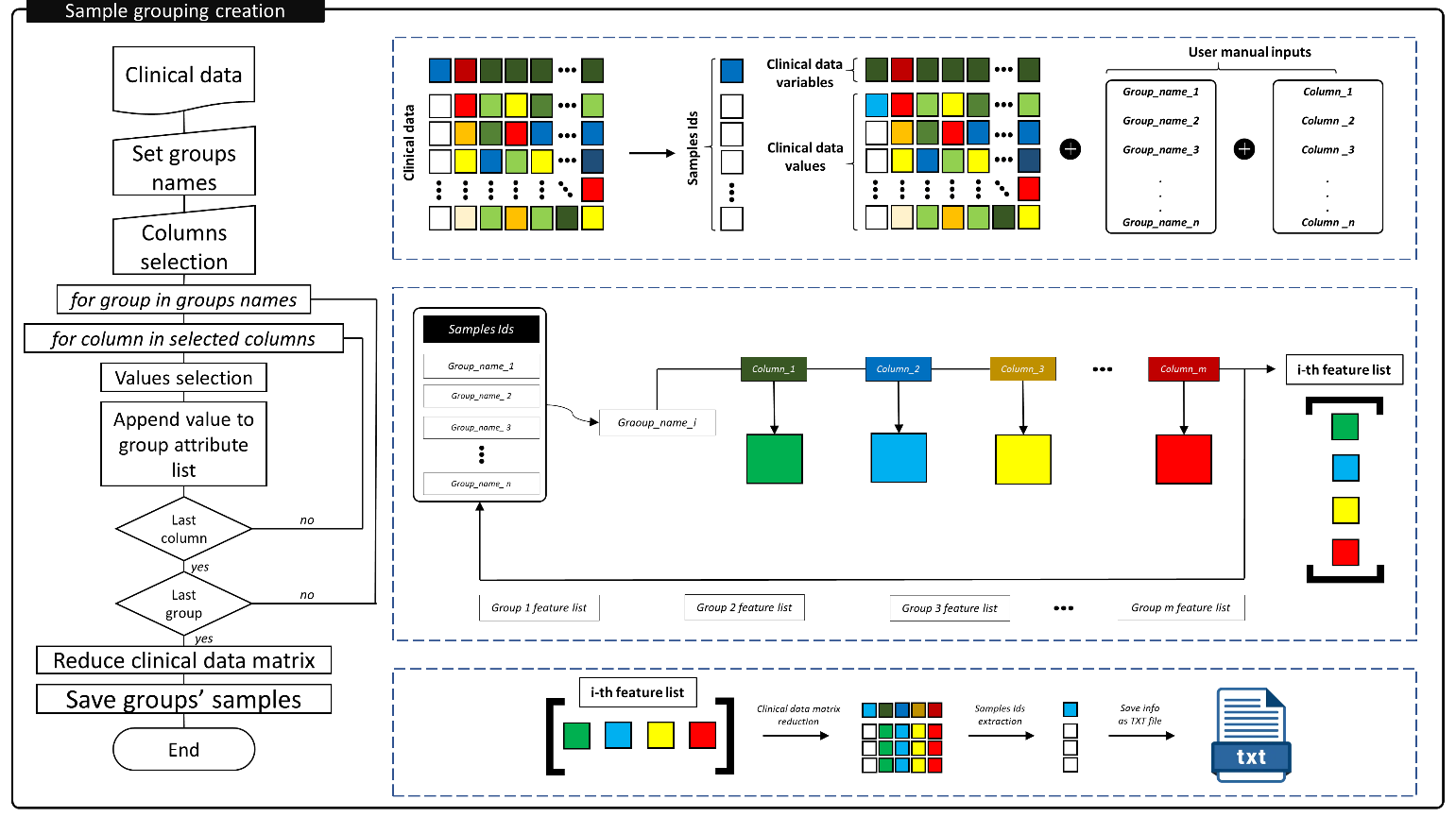


*Supplementary Figure 3 provides an overview of the second script in FunTB, detailing its workflow. The illustration depicts the script's progression from the clinical data table. It iterates through each selected column, presenting the user with possible values to choose from. Upon selection, the script reduces the dataframe for the next iteration until reaching the final column. Subsequently, the script saves a TXT file containing the sample IDs that exhibit every selected value. This iterative process is repeated for a desired number of groups.*

1. **Phenotype centric Network.**

In the final step of the FunTB process, the algorithm takes several input parameters, including the variation dictionary file and a set of sample lists from different phenotypic groups. Each list is processed to extract gene-related information. For every gene found, the algorithm stores it in a dictionary where the genes serve as the main keys. The corresponding values are the positions within those genes that exhibit at least one alteration. If a gene is found in multiple samples, the algorithm updates the dictionary values. This update may include adding new positions or increasing the frequency count for positions that have already been registered.

After processing all sample lists, each sample's list of IDs is stored in an array. This array serves as a reference for later steps when assigning names and linking nodes in the network. The next step involves performing all possible pairwise comparisons between the groups. This comparison eliminates genes that are shared between groups, keeping only those positions unique to each phenotypic cluster. After this comparison, there are two types of gene nodes: 1-degree nodes, which appear in only one phenotypic group, and n-degree nodes (where n > 1), which appear in multiple groups but with different mutation positions in each group. These n-degree genes will be linked to multiple phenotype nodes.

Next, the algorithm assigns properties to the nodes, such as color and size. Colors are assigned randomly from a generated list of colours. This list is created using a lambda function, which, when provided with the number of groups to analyse, generates a list of n+1 colors. Each color corresponds to a specific group, and any gene present in multiple groups is assigned the final color. The size of each node is determined by the Comprehensive Alteration Impact Score (CAIS). The CAIS is a weighted combination of three metrics: Alteration Density Score (ADS), Dominant Altered Gene Score (DAGS), and Cluster Diversity Alteration Score (CDAS).

The Alteration Density Score (ADS) is calculated by dividing the total number of alterations by the total number of variable positions, reflecting the density of alterations in the gene. The Cluster Diversity Alteration Score (CDAS) measures how alterations are distributed across different genes within the cluster, indicating which genes have a higher alteration rate. The Dominant Altered Gene Score (DAGS) is computed as the ratio of the maximum number of alterations in a gene to the total number of alterations for that gene. These three scores are then combined, with the CAIS score serving as the basis for determining the node size.

Additional parameters are also required for the process:

- Network_name: Specifies the names for the output files.
- Pareto_frontiers: Defines the number of top pareto fronts to include in the final network.
- Alpha: The weight factor for the Alteration Density Score (float).
- Beta: The weight factor for the Cluster Diversity Alteration Score (float).
- Gamma: The weight factor for the Dominant Altered Gene Score (float).

These parameters, together with the CAIS, ensure that the network construction accurately reflects the variations and relationships between genes and phenotypic groups, with each gene node properly sized and coloured based on its significance in the network.


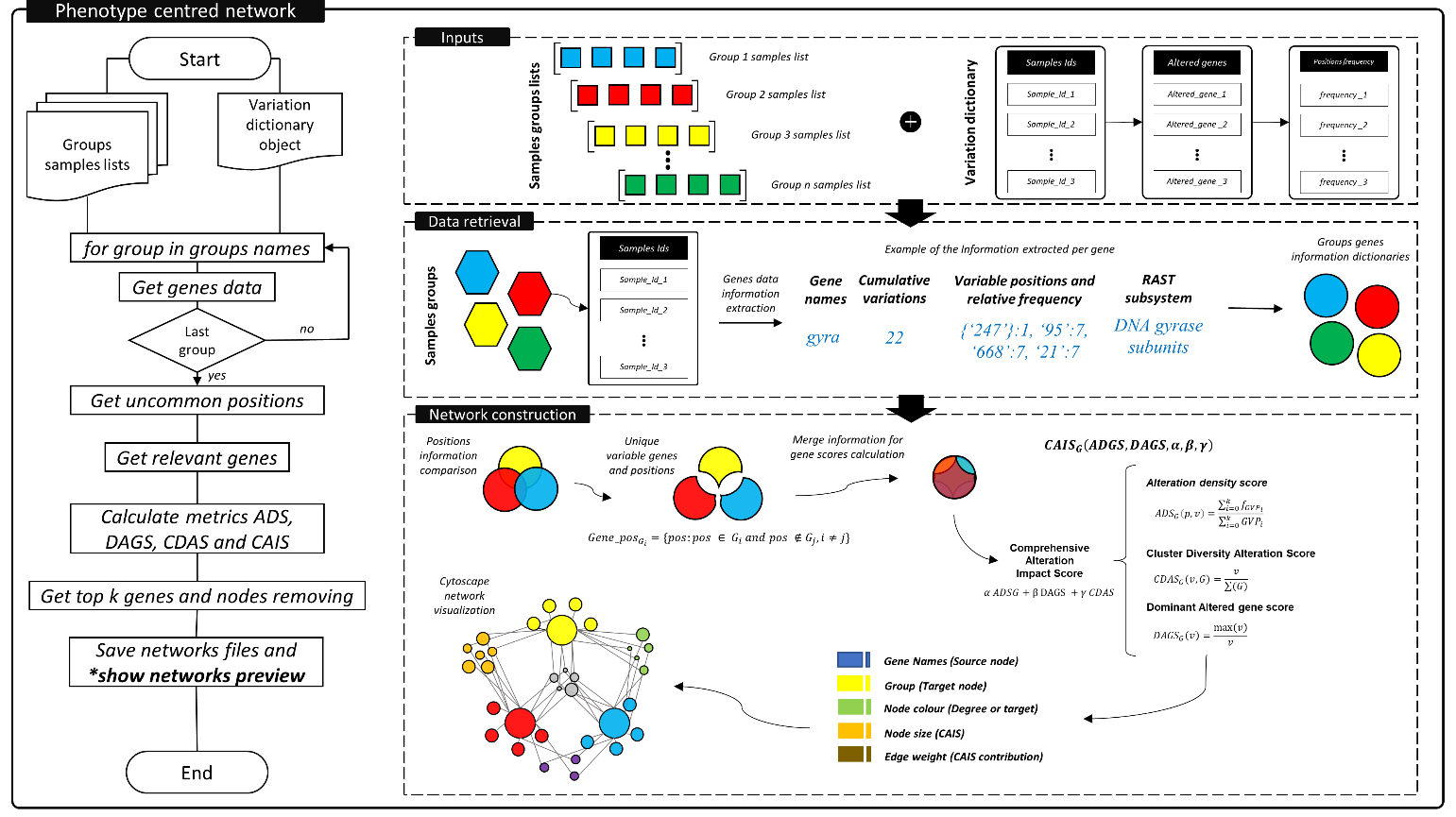


Supplementary Figure 4, FunTB Script number three, focused on network structuration, we could see on the left the flowchart diagram of the algorithm and on the right the schematic representation of it. First, it takes as input the two previously generated outputs (Variation dictionary and Samples lists) as we observe on the scheme at the top. Then from each of the list we took the ids and extract gene information, summarizing all the positions and found frequencies. Using this information, four alteration metrics are calculated (**ADS, CDAS, DAGS and CAIS**), CAIS metric is used then to assign node size, finally the Networkx object is saved within three different XML-Network file formats (GRAPHML, GML, GEXF).

**Note 3 – Algorithmic Implementation.**

To provide a clear understanding of the algorithm used to achieve this process, Supplementary Table 1 offers an overview of the variation dictionary's structure, highlighting the hierarchical organization of data at each level. This includes samples as primary keys, genes as nested keys, and the detailed tracking of altered positions and their associated frequencies. The accompanying pseudocode outlines the systematic steps involved in constructing this dictionary, ensuring transparency and reproducibility.

| **Algorithm 1: funtb variation dictionary creation** | | | | | | | |
| --- | --- | --- | --- | --- | --- | --- | --- |
|  | ***Input:*** *VCF MTBSeq output* | | | | | | |
|  | ***Output:*** *Dictionary comprising by genes and their associated frequency variation per altered position.* | | | | | | |
| **1** | ***Initialization of variables:*** *Declare* ***non_synonym_variations*** *as an empty dictionary* | | | | | | |
| **2** | ***for each*** *sample in* *samples* ***do*** *// Gets information about every sample* | | | | | | |
| **3** |  | ***non_synonym_variations ←*** *{}* *//Add a new key to the catalogue dictionary* | | | | | |
| **4** |  | ***for*** *row in nsv_df rows* ***do*** *//Enables the complete access to slice DF information* | | | | | |
| **5** |  |  | ***variation ←*** *variationPosition (nsv_df [row, sample])* | | | | |
| **6** |  |  | ***If*** *variation!* ***=*** *none* ***/****/ Assess the presence of a mutation* | | | | |
| **7** |  |  |  | ***gene ←*** *nsv_df [GeneName][row]* | | | |
| **8** |  |  |  | ***if*** *gene* ***not in*** *non_synonym_variations[sample]* | | | |
| **9** |  |  |  |  | | *Create a new entry for gene in non_synonym_variations[sample]* | |
| **10** |  |  |  |  | | *Initialize* ***total_variations*** *for the* ***gene*** *as* ***0*** | |
| **11** |  |  |  |  | | *Initialize* ***variation_positions*** *for the* ***gene*** *as an* ***empty dictionary*** | |
| **12** |  |  |  | ***if*** *variation* ***not in*** *non_synonym_variations[sample][gene][variation_positions]* | | | |
| **13** |  |  |  | |  | ***non_synonym_variations[sample][gene][‘total_varitions’] +=*** *1* | |
| **14** |  |  |  | |  | ***non_synonym_variations[sample][gene][‘variation_positions’][variation] =*** *1* | |
| **15** |  |  |  | ***else*** | | | |
| **16** |  |  |  |  | | Increment the frequency of variation in variation_positions by 1 | |
| **17** |  |  |  |  | |  |  |
| **18** |  | ***end*** |  |  | |  |  |
| **19** | ***end*** |  |  |  | |  |  |

Supplementary Table 1. FunTB Variation dictionary creation algorithm. This table provides a detailed breakdown of the dictionary's hierarchy, showcasing the relationship between samples, genes, variation positions, and associated frequencies. Each sample serves as the top-level key, followed by genes as nested keys. Within each gene, two components are stored: the total number of variations and a sub-dictionary containing specific altered positions and their respective frequencies.

Algorithm 2 facilitates the grouping of samples based on user-defined phenotypic characteristics from a structured metadata file. This enables targeted analysis by selecting specific subsets of samples that meet predefined conditions.

User can apply multiple filtering conditions based on the available metadata columns. These conditions may include:

- **Phenotypic resistance status** (e.g., selecting only resistant, susceptible, or intermediate isolates for specific antibiotic).
- **Species classification** (e.g., filtering *Mycobacterium tuberculosis complex* species).
- **Clinical metadata** (e.g., geographic region, patient history, or strain lineage, if available dataset).
- **Genotypic features** (e.g., presence of specific resistance mutations or gene deletions).

Filtering conditions are applied using an **AND** logic, meaning that a sample must meet all specified criteria to be included in the output list.

The metadata file should be provided as a **CSV** file containing mandatorily the column corresponding to **Sample ID** (unique identifier), the rest of the columns could hold any additional metadata. Supplementary Table 2 offers an overview of the sample list creation process.

**Algorithm 2: Samples List Creation.**

| **Algorithm 2: Samples lists creation** | | | | | | |
| --- | --- | --- | --- | --- | --- | --- |
|  | ***Input:*** *Clinical data file (.csv), Selected columns for filtering.* | | | | | |
|  | ***Output:*** *Grouped sample ID lists in text files.* | | | | | |
| **1** | ***Initialization*** *working directory (FunTB_dir) and load clinical data file into a dataframe* | | | | | |
| **2** | *Prompt the user to define the number of groups (****number_of_groups****)* | | | | | |
| **3** | *Get group names from the user (****Groups_names****)* | | | | | |
| **4** |  | ***for each*** *group* ***in*** *Groups_names* ***do:*** | | | | |
| **5** |  |  | *Ask the user to assign a name.* | | | |
| **6** |  |  | *Initialize an empty dictionary for filter conditions.* | | | |
| **7** |  |  | ***for each*** *column in* ***Selected_columns*** *do:* | | | |
| **8** |  |  |  | *Repeat until a valid filter condition is provided:* | | |
| **9** |  |  |  | *Display all unique values in that column.* | | |
| **10** |  |  |  |  | *Prompt the user to select a valid value.* | |
| **11** |  |  |  |  | *Store the selected value in the filter conditions.* | |
| **12** |  |  |  |  |  | |
| **13** |  |  | *Apply the filter conditions to the dataframe.* | | | |
| **14** |  |  | *Extract the sample IDs* *from the* ***filtered dataframe.*** | | | |
| **15** |  |  | *Save the filtered sample IDs to a text file.* | | | |
| **16** |  | ***end*** |  |  |  |  |
| **17** | ***end*** |  |  |  |  |  |

Supplementary Table 2. The algorithm facilitates the grouping of samples based on user-defined phenotypic characteristics from a structured metadata file. The algorithm begins by loading a CSV file where the first column contains unique sample identifiers, and subsequent columns store relevant phenotypic and genotypic attributes. The user specifies the number of groups and assigns a name to each. For every group, the user selects metadata columns to filter and chooses values from the available options. The algorithm then applies these conditions to extract matching samples. The filtered sample IDs are saved in text files, with each file named according to the corresponding group. These outputs can be used in further analyses, including statistical comparisons and machine learning applications. The input metadata file must follow a structured format to ensure consistency, and a sample dataset will be provided for reference.

**Step-by-Step example**

A researcher wants to extract a list of rifampicin-resistant *M. tuberculosis isolates for further analysis:*

**User input:**

- Number of groups: 1
- Group name: Rifampicin resistance

**Selection of filtering conditions.**

- **Metadata column:** Species 🡪 Select *M. tuberculosis.*
- **Metadata column**: Resistance status 🡪 Select *Resistance.*
- **Metadata column:** Antibiotic 🡪 Select *Rifampicin*

**Data processing:**

- The algorithm filters dataset based on selections.
- Only samples that match all three criteria are included in the final list.

**Output**

- A text file named *Rifampicin_Resistant.txt,* containing the sample IDs of isolates that meet the defined conditions*.*

Algorithm 3, tackles the construction of a variation graph that represents the relationship between genes and phenotypic groups. The process begins by combining the sample lists for each phenotypic group, followed by the extraction of relevant gene variation data for both the interest and background samples. Signature genes are identified by comparing the variation data, and non-PE and non-PPE genes are filtered out. Using this information, the algorithm generates a network where nodes represent genes and edges reflect their association with specific groups. Node attributes, such as size and colour, are assigned based on the density of gene variations and the group affiliations. This network visualization enables an intuitive understanding of how genetic variations correlate with phenotypic traits, providing valuable insights for further analysis

| **Algorithm 3: funtb network constructions** | | | | | | | |
| --- | --- | --- | --- | --- | --- | --- | --- |
|  | ***Input:*** *Groups samples lists, background samples list, non_synonym_variations_dictionary* | | | | | | |
|  | ***Output:*** *A series of XML-based file formats for graphs with gene-phenotypic-group relation* | | | | | | |
| **1** | ***Initialization of variables:*** *Declare the* ***network*** *variable as an empty data frame* | | | | | | |
| **2** | ***samples ←*** *concatenate(group_samples_lists)* | | | | | | |
| **3** | ***interest_genes_data ←*** *GenesData(samples, non_synonym_variations_dictionary)* | | | | | | |
| **4** | ***background_genes_data ←*** *GenesData(background_samples_list, non_synonym_variations_dictionary)* | | | | | | |
| **5** | ***signatures ←*** *GetSigantures(interest_genes_data, background_genes_data)* | | | | | | |
| **6** | ***signatures ←*** *non_ppe_pp_filter(signatures)* | | | | | | |
| **7** | ***groups ←*** *permutations(samples)* | | | | | | |
| **7** | ***groups_colors ←*** *permutations(groups.length())* | | | | | | |
| **6** |  | ***for*** *node in variation_graph* ***do*** *//Enables the complete access to slice DF information* | | | | | |
| **8** |  |  | ***If*** *node* ***in*** *groups* ***/****/ Assess if mutation has been added to gene info* | | | | |
| **9** |  |  |  | *variation_graph.nodes[node][size] = max(signatures[density_variation_score]) * 5* | | | |
| **10** |  |  |  | *variation_graph.nodes[node][color] = group_colors_dictionary[node]* | | | |
| **11** |  |  | *else* | | | | |
| **12** |  |  |  | | *variation_graph.nodes[node][size] = signatures[gen][density_variation_score]* | | |
| **13** |  |  |  | ***if*** v*ariation_graph. degree[node] == 1* | | | |
| **14** |  |  |  |  | | ***variation ← bfs(nodes, group_colors)*** | |
| **15** |  |  |  | ***else*** | | | |
| **17** |  |  |  |  | | ***variation ← degree(node)*** | |
| **18** |  | ***end*** |  |  | |  |  |

Supplementary Table 3 This algorithm describes the process of constructing gene-phenotypic-group relation networks. It takes as input the sample lists for each group, the background sample list, and the non-synonymous variation dictionary. The algorithm generates a series of XML-based graph files, with each graph depicting the relationships between genes and phenotypic groups. The network construction incorporates gene variation densities and group-specific attributes to visualize gene-phenotype associations.

**Note 4 – Metrics’ Equations and Interpretation.**

FunTB constructs a phenotype-centered network with two distinct node types to elucidate

in which each node represents either a gene with altered positions or a phenotype-centred "ego-node." Genes that retain at least one distinct mutation position are added to the network as nodes, positioned around and linked to the ego-node associated with their specific phenotypic group. This phenotypic-centred network structure provides a clear visualization of variation distributions and the relationships among genes across different groups.

**Alteration density score (ADS).** Equation (1) Measures the density of alterations within a gene by calculating the ratio of the total number of variations to present within the gene and the total number of positions.

$$ADSG\left( {f_{GVp},G}_{vp} \right)=\frac{\sum_{i=0}^{k} f_{GVP_{i}}}{G_{vp}} (1)$$

Where,

- **ADSG(**$\boldsymbol{f}_{\boldsymbol{GV}\boldsymbol{P}_{\boldsymbol{i}}}$**,** $\boldsymbol{GV}\boldsymbol{P}_{\boldsymbol{i}}$**)**: Represents the Alteration Density Score for gene G at all mutated position *p* and variation *v*.
- $\boldsymbol{f}_{\boldsymbol{GV}\boldsymbol{P}_{\boldsymbol{i}}}$: Frequency of the presence of a gene variation at position i.

- $\boldsymbol{G}_{\boldsymbol{vp}}$**​**: Total altered gene positions within gene G.

*ADS* measures the density of alterations within a gene by calculating the ratio of the total frequency of variations across all mutated positions to the total number of altered positions in that gene. In biological terms, a low ADS indicates that, although a gene may harbour mutations, these alterations are concentrated in specific, functionally critical regions (e.g., drug-binding pockets). This concentration is often more biologically relevant—for example, mutations clustered in the rifampicin resistance-determining region (RRDR) of rpoB are functionally significant. Conversely, a high ADS suggests widespread mutations across the gene, which can include non-specific background alterations that may not contribute directly to phenotype.

**Dominant Altered gene score (DAGS).** Equation (2) Evaluates he dominance of gene alterations by calculating the ratio of the maximum frequency of a gene variation to the total number of altered gene positions. This score identifies genes with a prevalent alteration pattern. A high **DAGS** signifies that a specific mutation is particularly prevalent, suggesting it may play a critical role in driving phenotypic change.

$$DAGS\left( \boldsymbol{f}_{\boldsymbol{GV}\boldsymbol{P}_{\boldsymbol{i}}}\mathbf{,}\boldsymbol{GVP} \right)=\frac{\max\left( \boldsymbol{f}_{\boldsymbol{GV}\boldsymbol{P}_{\boldsymbol{i}}} \right)}{G_{vp}} (2)$$

Where:

- **DAGS (**$\boldsymbol{f}_{\boldsymbol{GV}\boldsymbol{P}_{\boldsymbol{i}}}$**,** $\boldsymbol{GVP}$**)**: Represents the Dominant Altered gene Score for gene G.
- $\max\left( \boldsymbol{f}_{\boldsymbol{GV}\boldsymbol{P}_{\boldsymbol{i}}} \right)$**:** Maximum value of frequency of a gene variation at position i.
- $\boldsymbol{G}_{\boldsymbol{vp}}\boldsymbol{:}$ Total altered gene positions within gene G.

**Cluster Diversity Alteration Score (CDAS).** Equation (3) Assesses how gene alterations are distributed across different phenotypic groups. It is defined as the ratio of the total variation frequency in a gene to the number of altered genes within the corresponding phenotypic cluster. A high CDAS indicates that a gene’s mutations are spread across multiple phenotypic groups, which might suggest a broader role in adaptation or, if excessively high, may reflect non-specific background variation. A low CDAS implies that the mutations are more confined to a specific phenotype, thereby enhancing the signal of phenotype-specific alterations.

$$CDAS\left( \sum_{\boldsymbol{i=0}}^{\boldsymbol{k}} \boldsymbol{f}_{\boldsymbol{GV}\boldsymbol{P}_{\boldsymbol{i}}}\mathbf{,}\sum\boldsymbol{(G)} \right)=\frac{\sum_{i=0}^{k} f_{GVP_{i}}}{\sum(G)} (3)$$

Where:

- **CDAS (**$\sum_{\boldsymbol{i=0}}^{\boldsymbol{k}} \boldsymbol{f}_{\boldsymbol{GV}\boldsymbol{P}_{\boldsymbol{i}}}$**,** $\sum\boldsymbol{(G)}$**)**: Represents the Cluster Diversity Alteration Score for gene 𝐺.
- $\sum_{\boldsymbol{i=0}}^{\boldsymbol{k}} \boldsymbol{f}_{\boldsymbol{GV}\boldsymbol{P}_{\boldsymbol{i}}}$**:** Total frequency of variations across all positions i for gene G.
- $\sum\boldsymbol{(G)}\boldsymbol{:}$ Total altered genes within belong phenotypic cluster.

**Comprehensive Alteration Impact Score (CAIS).** Equation (4) Integrates multiple aspects of gene alterations using a weighted combination of the Alteration Density Score, Dominant Altered Gene Score, and Cluster Diversity Alteration Score.

$$CAIS\left( ADGS, DAGS,CDAS,\alpha,\beta,\gamma\right)=\alpha ADSG+\beta DAGS +\gamma CDAS (4)$$

Where:

- **CAIS (*ADSG, DAGS, α, β, γ*)**: Represents the Comprehensive Alteration Impact Score for a gene.
- **α, β, γ:** Weights assigned to each score, reflecting their relative importance.
- **ADSG:** Alteration Density Score, capturing the frequency of variations.
- **DAGS:** Dominant Altered Gene Score, indicating the dominance of specific variations.
- **CDAS:** Cluster Diversity Alteration Score, assessing variation distribution across phenotypic groups.

These metrics provide a comprehensive way to evaluate the impact and relevance of gene alterations. These scores can be used to rank genes or prioritize further analysis.

**Note 5 – Statistical validation.**

The statistical analysis conducted evaluates the significance of the optimized parameter set (α=0.9386, β=0.0008, γ=0.0607) in comparison to random parameter configurations. This analysis is designed to validate whether the optimized parameters yield a fitness score significantly different from those generated by random parameter assignments outside a predefined buffer zone.

To perform this analysis, a buffer zone of ±5% around the optimal α and β values was defined, ensuring that the random samples excluded configurations near the optimal set. A total of 1,000 random parameter combinations were generated, adhering to the constraint α+β+γ=1 and maintaining γ≥0. For each random configuration, the fitness score was calculated using the FunTB tool, capturing the performance of the random parameters.

The optimized fitness score was directly compared to the distribution of fitness scores from the random configurations using a one-sample t-test. This statistical test assesses whether the mean of the random scores significantly deviates from the optimized fitness score. The resulting t-statistic and p-value provide a quantitative measure of this difference. A p-value below 0.05 indicates statistical significance, suggesting that the optimized parameters yield a fitness score meaningfully different from those derived from random sampling.

In the statistical analysis conducted, the p-value obtained for both tests were 0, indicating extremely strong evidence that the optimized parameter set (α = 0.9386, β = 0.0008, γ = 0.0607) produces fitness scores significantly different from those generated by random parameter combinations outside the buffer zone.

This result suggests that the optimized parameters identified through the optimization process are highly effective in enhancing the fitness score, as compared to randomly selected parameter sets. A p-value of 0.00001 effectively rejects the null hypothesis of no difference, highlighting that the optimization method successfully identified a distinctively superior parameter configuration.

Such findings emphasize the robustness and precision of the optimization process in fine-tuning the parameters to achieve the desired outcomes.

**Note 6 – Pareto front strategy to identify outstanding genes.**

To prioritize genes for visualization in our final network, we implemented a multi-objective Pareto optimization process that incorporates four metrics: *ADS*, *DAGS*, *CDAS*, and *CAIS*. We minimized *ADS* to reduce the prevalence of non-specific, widespread alterations, while maximizing DAGS, CDAS, and CAIS to emphasize dominant mutations, enhance phenotype specificity, and integrate these factors into a single comprehensive metric. To define the optimization criteria, we analysed the CRyPTIC database [1], focusing on samples resistant to isoniazid and rifampicin, as well as sensitive samples. Using FunTB, we identified genes with mutations previously linked to drug resistance, examined the distribution of each metric, and established our optimization parameters accordingly.

The Pareto optimization strategy in FuNTB is built on three biologically grounded metrics integrated into CAIS. Genes with high ADS, such as those in the PE/PPE family, often accumulate widespread mutations that are not directly linked to phenotypic adaptation. By minimizing ADS, our approach emphasizes genes where mutations are concentrated in functionally significant regions, such as drug-binding pockets. For example, in rpoB, which is associated with rifampicin resistance, most mutations cluster within the rifampicin resistance-determining region (RRDR), an 81-bp hotspot that reflects biologically meaningful mutational patterns critical for antibiotic resistance [2]. Additionally, the focus on genes with high CDAS enhances phenotype specificity by identifying mutations enriched in specific groups—such as pncA mutations in pyrazinamide-resistant strains—thus reducing false positives from shared or background mutations [3]. High *DAGS* further highlights genes with dominant, recurrent mutations validated to drive phenotypic changes; for instance, rpoB S450L accounts for over 60% of rifampicin resistance mutations [4], while *katG* S315T is present in approximately 70% of isoniazid-resistant isolates [5]. These dominant mutations reinforce known resistance mechanisms and contribute to the reproducibility of our findings.

To achieve optimal visualization, we calibrated the weighting coefficients (𝛼, β, and γ) for CAIS using Grid Search, Randomized Search, and Bayesian Search on CRyPTIC data. Our goal was to capture key resistance genes (e.g., katG, rpoB, pncA) while minimizing the number of Pareto fronts required. The objective function is defined by the fitness score (Equation (5)), which we aimed to maximize.

$$f_{s}=0.5 G_{{PF}_{1}}+0.3G_{{PF}_{2}}+0.2G_{{PF}_{3}}+0.07 G_{{PF}_{4}}+0.03 G_{{PF}_{5}} (5)$$

Where:

- $\boldsymbol{f}_{\boldsymbol{s}}$**:** Fitness score, quantifies how effectively a set of coefficients prioritizes canonical genes in early pareto fronts.
- $\boldsymbol{Fixed weights (}\boldsymbol{w}_{\boldsymbol{i}}\boldsymbol{)}$**:** Represents weight of number of genes present within ith Pareto Front and prioritize PF1 (highest weight) while penalizing genes in later fronts (0.5, 0.3, 0.2, 0.07, 0.03).
- $\boldsymbol{G}_{\boldsymbol{PF}_{\boldsymbol{i}}}$**:** Number of predefined genes of interest (GoI) in the i-th Pareto front.

The optimization workflow started with a parameter search step constrain of (𝛼 + β + γ = 1).:

- ***Grid search****:* Tested 10000 predefined (𝛼, β, and γ) combinations exhaustively.
- ***Randomized search:*** Sampled 1000 random coefficients.
- ***Bayesian search*:** Used probabilistic modelling to iteratively refine coefficients over 50 evaluations, balancing exploration and exploitation.

Then we performed an evaluation process for each method, coefficients were ranked by their fitness score, as results we got that: Bayesian Search achieved the highest fitness score (0.45), concentrating 9 GoI in PF1 (e.g., rpoB, katG) and 3 in PF2, with none in later fronts (Table 1). This outperformed Grid Search (fitness = 0.43, 7 GoI in PF1) and Randomized Search (fitness = 0.42, 7 GoI in PF1).

Table 1 Optimization Methods Performance Comparison

| Optimization Method | alpha | Beta | Gamma | Best Fitness Score | Number of Evaluations | Genes of Interest Distribution |
| --- | --- | --- | --- | --- | --- | --- |
| Grid search | 0.9000 | 0.0000 | 0.1000 | 0.43 | 1000 | [7, 2, 1, 0, 0] |
| Randomized search | 0.1300 | 0.8100 | 0.0500 | 0.42 | 100 | [7, 3, 0, 0, 0] |
| Bayesian search | 0.9400 | 0.0007 | 0.0600 | 0.45 | 50 | [9, 3, 0, 0, 0] |

The Bayesian Search method yielded the best fitness score of 0.45, prioritizing nine genes in the first Pareto front (PF1) and three genes in the second (PF2), with no genes in the remaining fronts. This outcome demonstrated the effectiveness of Bayesian Search in concentrating key genes within the top Pareto fronts, ensuring a streamlined visualization and meaningful insights.


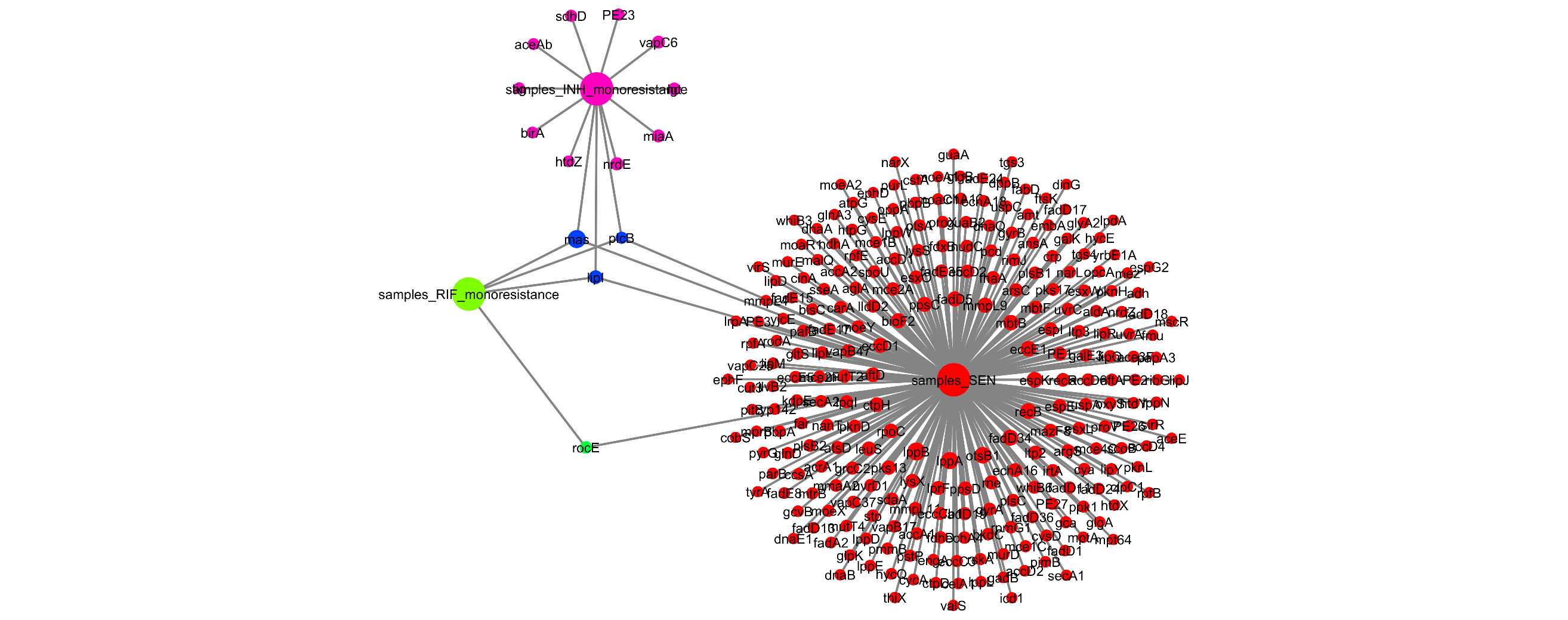


Supplementary figure 5, Final network of gene alterations in Rifampicin-resistant, Isoniazid-resistant, and sensitive Mycobacterium tuberculosis samples from the CRyPTIC dataset. This visualization captures the relationships between genes and phenotypic groups, with node size reflecting CAIS (gene impact) and edges denoting observed alterations. The colour scheme employs three primary colours to represent genes exclusively associated with a single phenotype: green for Rifampicin-resistant, blue for Isoniazid-resistant, and red for Sensitive. Some gene nodes appear in distinct colours, indicating they harbour unique altered positions across multiple phenotypic groups. These were assigned separate colours to distinguish them from genes strictly linked to one phenotype, improving clarity and highlighting complex resistance patterns.

**Note 7 – Network Visualization.**

| Gene | ADS | DAGS | CDAS | CAIS |
| --- | --- | --- | --- | --- |
| proV | 0.0436 | 0.0142 | 0.0834 | 0.046 |
| xthA | 0.0407 | 0.0175 | 0.0687 | 0.0424 |
| pks5 | 0.0577 | 0.0027 | 0.708 | 0.0971 |
| kdpA | 0.0443 | 0.004 | 0.1939 | 0.0533 |
| mmpL11 | 0.0947 | 0.0218 | 0.1534 | 0.0982 |
| mtr | 0.0862 | 0.0557 | 0.1178 | 0.0881 |
| rodA | 0.0584 | 0.0166 | 0.1521 | 0.0641 |
| accD5 | 0.0647 | 0.0307 | 0.0761 | 0.0654 |
| fadD15 | 0.0678 | 0.0139 | 0.2221 | 0.0771 |
| mmuM | 0.0407 | 0.0175 | 0.0687 | 0.0424 |
| adhE1 | 0.1612 | 0.0857 | 0.1313 | 0.1593 |
| proX | 0.055 | 0.0147 | 0.1252 | 0.0592 |
| corA | 0.0299 | 0.0077 | 0.0626 | 0.0319 |
| eccCb1 | 0.0856 | 0.0237 | 0.2012 | 0.0926 |
| lprO | 0.1393 | 0.0557 | 0.1178 | 0.1379 |
| oppB | 0.0358 | 0.0137 | 0.0626 | 0.0374 |
| fadE18 | 0.0299 | 0.0057 | 0.1178 | 0.0352 |
| eccE2 | 0.1003 | 0.0249 | 0.2638 | 0.1102 |
| fadE20 | 0.1493 | 0.0594 | 0.1043 | 0.1465 |
| grcC2 | 0.0784 | 0.0332 | 0.1043 | 0.0799 |
| devR | 0.0299 | 0.0557 | 0.0074 | 0.0286 |
| mrsA | 0.1384 | 0.0502 | 0.0687 | 0.1341 |
| ribH | 0.0697 | 0.0757 | 0.0147 | 0.0664 |
| clpC1 | 0.0505 | 0.0096 | 0.173 | 0.0579 |
| rocA | 0.0597 | 0.0157 | 0.1178 | 0.0632 |
| espB | 0.0985 | 0.0332 | 0.2699 | 0.1088 |
| bkdC | 0.039 | 0.0142 | 0.0834 | 0.0417 |
| atsD | 0.0637 | 0.0177 | 0.2012 | 0.072 |
| mshC | 0.0611 | 0.0128 | 0.1387 | 0.0658 |
| cydD | 0.0624 | 0.0284 | 0.0687 | 0.0628 |
| vapB19 | 0.0896 | 0.0642 | 0.0417 | 0.0867 |
| pgi | 0.0576 | 0.0086 | 0.1865 | 0.0654 |
| fadD2 | 0.0627 | 0.0107 | 0.1313 | 0.0668 |
| dgt | 0.0537 | 0.0317 | 0.0626 | 0.0542 |
| mrp | 0.0336 | 0.0107 | 0.1043 | 0.0379 |
| rpoB | 0.0757 | 0.0152 | 0.2908 | 0.0887 |
| lppA | 0.0657 | 0.0317 | 0.0626 | 0.0655 |
| bacA | 0.0384 | 0.0128 | 0.0896 | 0.0415 |
| fabG2 | 0.3085 | 0.1623 | 0.0552 | 0.293 |
| fadD9 | 0.0697 | 0.0157 | 0.3472 | 0.0865 |
| lpqK | 0.1335 | 0.0683 | 0.1252 | 0.1329 |
| cya | 0.0497 | 0.01 | 0.1387 | 0.0551 |
| glpQ1 | 0.1469 | 0.0509 | 0.1669 | 0.148 |
| ask | 0.0574 | 0.0234 | 0.0834 | 0.059 |
| galK | 0.0567 | 0.0107 | 0.1313 | 0.0612 |
| groEL1 | 0.0945 | 0.0457 | 0.0761 | 0.0933 |
| wag22 | 0.0992 | 0.0247 | 0.2074 | 0.1057 |
| lipN | 0.0738 | 0.0147 | 0.1252 | 0.0769 |
| pca | 0.0398 | 0.0037 | 0.2012 | 0.0496 |
| senX3 | 0.1095 | 0.0857 | 0.0761 | 0.1075 |
| aprB | 0.3284 | 0.2655 | 0.0209 | 0.3097 |
| mutA | 0.0299 | 0.0032 | 0.1043 | 0.0344 |
| etgB | 0.0426 | 0.0128 | 0.0896 | 0.0454 |
| desA3 | 0.1459 | 0.0823 | 0.1178 | 0.1441 |
| mazE9 | 0.0448 | 0.0407 | 0.0209 | 0.0433 |
| xseB | 0.1493 | 0.2355 | 0 | 0.1403 |
| moeY | 0.2186 | 0.0944 | 0.2074 | 0.2178 |
| gmhB | 0.0361 | 0.0052 | 0.1252 | 0.0415 |
| pstA2 | 0.1244 | 0.0807 | 0.0761 | 0.1214 |
| pks10 | 0.1379 | 0.0814 | 0.1387 | 0.1379 |
| lgt | 0.063 | 0.0157 | 0.1178 | 0.0663 |
| arsB1 | 0.0768 | 0.0257 | 0.0896 | 0.0775 |
| pcd | 0.0497 | 0.0117 | 0.0969 | 0.0525 |
| ssb | 0.1015 | 0.0677 | 0.027 | 0.097 |
| mmpL3 | 0.0702 | 0.0186 | 0.2282 | 0.0797 |
| ppiA | 0.0841 | 0.0393 | 0.0687 | 0.0831 |
| TB27.3 | 0.0736 | 0.0277 | 0.0969 | 0.075 |
| vapC17 | 0.0597 | 0.0557 | 0.0209 | 0.0573 |
| secY | 0.1625 | 0.129 | 0.0552 | 0.156 |
| metB | 0.0407 | 0.0175 | 0.0687 | 0.0424 |
| dapF | 0.0597 | 0.0457 | 0.0344 | 0.0582 |
| mmpL1 | 0.0767 | 0.0169 | 0.3472 | 0.0931 |
| serC | 0.0554 | 0.0385 | 0.0417 | 0.0546 |
| glmS | 0.0369 | 0.0098 | 0.1104 | 0.0413 |
| narL | 0.0497 | 0.0357 | 0.0344 | 0.0488 |
| fadD6 | 0.0579 | 0.0169 | 0.1104 | 0.0611 |
| ftsK | 0.0862 | 0.0324 | 0.1178 | 0.0881 |
| cdsA | 0.0299 | 0.009 | 0.0552 | 0.0314 |
| rplX | 0.0299 | 0.0357 | 0.0135 | 0.0289 |
| fdxD | 0.1791 | 0.1156 | 0.0221 | 0.1695 |
| pepD | 0.0448 | 0.0107 | 0.1043 | 0.0484 |
| sigJ | 0.0299 | 0.0049 | 0.0834 | 0.0331 |
| esxA | 0.0299 | 0.0128 | 0.0417 | 0.0306 |
| eccD2 | 0.0382 | 0.0013 | 0.292 | 0.0536 |
| menD | 0.0941 | 0.0511 | 0.0834 | 0.0934 |
| rpsP | 0.1663 | 0.1499 | 0.0417 | 0.1587 |
| kstD | 0.0776 | 0.0317 | 0.1325 | 0.0809 |
| fadD3 | 0.0448 | 0.0157 | 0.0761 | 0.0467 |
| vapC44 | 0.097 | 0.0482 | 0.0479 | 0.094 |
| TB18.5 | 0.1119 | 0.0482 | 0.0479 | 0.108 |
| ppsC | 0.0678 | 0.0044 | 0.3742 | 0.0863 |
| modC | 0.085 | 0.0418 | 0.0834 | 0.0849 |
| proA | 0.057 | 0.023 | 0.0687 | 0.0577 |
| mmpL10 | 0.0641 | 0.0085 | 0.319 | 0.0795 |
| bpoB | 0.1004 | 0.0611 | 0.0687 | 0.0984 |
| pstS1 | 0.1147 | 0.0494 | 0.1252 | 0.1153 |
| glgA | 0.135 | 0.0614 | 0.1387 | 0.1352 |
| ctpG | 0.0382 | 0.0013 | 0.2908 | 0.0535 |
| aroF | 0.0299 | 0.0066 | 0.0687 | 0.0322 |
| eccC5 | 0.0461 | 0.0066 | 0.146 | 0.0521 |
| fprB | 0.0617 | 0.0197 | 0.0969 | 0.0638 |
| esxK | 0.1493 | 0.1456 | 0.0209 | 0.1415 |
| cydC | 0.0464 | 0.009 | 0.1178 | 0.0507 |
| cyp125 | 0.063 | 0.0157 | 0.1178 | 0.0663 |
| ahpD | 0.0299 | 0.0257 | 0.0221 | 0.0294 |
| vapB4 | 0.1493 | 0.1328 | 0.0417 | 0.1428 |
| pyrR | 0.4179 | 0.4154 | 0.0209 | 0.3938 |
| lpqU | 0.0299 | 0.0157 | 0.0344 | 0.0302 |
| truB | 0.0697 | 0.0557 | 0.0344 | 0.0675 |
| vapC36 | 0.0448 | 0.0257 | 0.0479 | 0.045 |
| pgsA2 | 0.1493 | 0.1156 | 0.0552 | 0.1436 |
| mbtA | 0.1119 | 0.0444 | 0.1043 | 0.1114 |
| hbhA | 0.0239 | 0.0077 | 0.0626 | 0.0262 |
| ppsB | 0.0541 | 0.0038 | 0.2491 | 0.0659 |
| gpgS | 0.0597 | 0.0557 | 0.0209 | 0.0573 |
| ligD | 0.0597 | 0.0096 | 0.173 | 0.0665 |
| drrC | 0.0299 | 0.0157 | 0.0344 | 0.0302 |
| ftsW | 0.1355 | 0.0511 | 0.0834 | 0.1323 |
| recN | 0.1919 | 0.1242 | 0.0896 | 0.1856 |
| cysE | 0.0829 | 0.0224 | 0.1178 | 0.085 |
| galU | 0.1254 | 0.0917 | 0.027 | 0.1194 |
| purN | 0.1834 | 0.1499 | 0.0417 | 0.1748 |
| ilvC | 0.0353 | 0.0121 | 0.0687 | 0.0373 |
| ung | 0.1134 | 0.0797 | 0.0626 | 0.1103 |
| fcoT | 0.0418 | 0.0197 | 0.0626 | 0.043 |
| fadE36 | 0.1407 | 0.0814 | 0.0896 | 0.1376 |
| bisC | 0.0433 | 0.0035 | 0.2074 | 0.0532 |
| aglA | 0.0566 | 0.0102 | 0.1939 | 0.0649 |
| fmu | 0.0929 | 0.0357 | 0.1178 | 0.0944 |
| fadE12 | 0.1387 | 0.0451 | 0.1104 | 0.1369 |
| lprF | 0.0516 | 0.0284 | 0.0687 | 0.0526 |
| whiB5 | 0.0678 | 0.0284 | 0.0687 | 0.0678 |
| adhD | 0.0522 | 0.0182 | 0.1043 | 0.0553 |
| fabG3 | 0.0469 | 0.03 | 0.0417 | 0.0466 |
| mmpS4 | 0.0299 | 0.0128 | 0.0417 | 0.0306 |
| vapC14 | 0.1493 | 0.0857 | 0.0209 | 0.1415 |
| infC | 0.0725 | 0.0471 | 0.0417 | 0.0706 |
| blaC | 0.0712 | 0.0234 | 0.0834 | 0.0719 |
| pimE | 0.0975 | 0.0517 | 0.0969 | 0.0974 |
| pstS2 | 0.055 | 0.0115 | 0.1252 | 0.0592 |
| dfp | 0.0412 | 0.0071 | 0.1387 | 0.0471 |
| mycP5 | 0.0544 | 0.0169 | 0.1104 | 0.0578 |
| hemK | 0.0461 | 0.0175 | 0.0687 | 0.0474 |
| cydA | 0.065 | 0.0169 | 0.1104 | 0.0677 |
| tatC | 0.0733 | 0.0284 | 0.0687 | 0.073 |
| otsB1 | 0.0929 | 0.0182 | 0.492 | 0.1171 |
| fadD13 | 0.1433 | 0.0976 | 0.2012 | 0.1468 |
| prcA | 0.0469 | 0.03 | 0.0417 | 0.0466 |
| hisI | 0.0299 | 0.0357 | 0.0147 | 0.029 |
| narH | 0.041 | 0.0107 | 0.1043 | 0.0448 |
| ndkA | 0.0697 | 0.0757 | 0.0135 | 0.0663 |
| lppR | 0.1493 | 0.0657 | 0.0761 | 0.1448 |
| fba | 0.0597 | 0.0257 | 0.0761 | 0.0607 |
| mycP1 | 0.0677 | 0.0037 | 0.2012 | 0.0757 |
| nadB | 0.124 | 0.0372 | 0.173 | 0.1269 |
| murG | 0.1294 | 0.0857 | 0.0761 | 0.1261 |
| fadD35 | 0.0409 | 0.009 | 0.1804 | 0.0493 |
| kdpD | 0.1023 | 0.034 | 0.319 | 0.1154 |
| lppT | 0.1493 | 0.2355 | 0 | 0.1403 |
| gid | 0.1678 | 0.0464 | 0.4871 | 0.1871 |
| ruvB | 0.0299 | 0.0066 | 0.0687 | 0.0322 |
| pks17 | 0.0948 | 0.0486 | 0.2282 | 0.1029 |
| ripA | 0.1125 | 0.0418 | 0.0834 | 0.1107 |
| fic | 0.0431 | 0.0224 | 0.0552 | 0.0438 |
| argD | 0.0615 | 0.0169 | 0.1104 | 0.0644 |
| kdpB | 0.0566 | 0.0081 | 0.1939 | 0.0649 |
| acpA | 0.0469 | 0.03 | 0.0417 | 0.0466 |
| pstS3 | 0.2239 | 0.1506 | 0.0761 | 0.2149 |
| gdh | 0.0674 | 0.0093 | 0.4233 | 0.0889 |
| gpsI | 0.0439 | 0.0098 | 0.1104 | 0.0479 |
| mfd | 0.052 | 0.009 | 0.3681 | 0.0711 |
| moxR3 | 0.0448 | 0.0257 | 0.0479 | 0.045 |
| vapC25 | 0.0597 | 0.0377 | 0.0626 | 0.0599 |
| pknG | 0.0924 | 0.02 | 0.2847 | 0.104 |
| vapC46 | 0.0213 | 0.0128 | 0.0417 | 0.0225 |
| nei | 0.0896 | 0.0414 | 0.1387 | 0.0925 |
| vapC23 | 0 | 0.0557 | 0.0074 | 0.0005 |
| vapB12 | 0.0299 | 0.0557 | 0.0074 | 0.0286 |
| dsbF | 0.0716 | 0.0437 | 0.0626 | 0.071 |
| thiL | 0.0763 | 0.0357 | 0.0552 | 0.075 |
| aceAb | 0.2201 | 0.1868 | 0.1043 | 0.213 |
| pheT | 0.0697 | 0.0126 | 0.2638 | 0.0814 |
| PE4 | 0.0987 | 0.0441 | 0.173 | 0.1032 |
| mbtM | 0.1407 | 0.0857 | 0.0896 | 0.1376 |
| ureC | 0.0896 | 0.027 | 0.1521 | 0.0933 |
| echA4 | 0.0564 | 0.0357 | 0.0552 | 0.0563 |
| oplA | 0.0725 | 0.0071 | 0.2847 | 0.0853 |
| rpoC | 0.061 | 0.0048 | 0.3117 | 0.0762 |
| vapB11 | 0.0299 | 0.0257 | 0.0209 | 0.0294 |
| fadD18 | 0.064 | 0.0214 | 0.0896 | 0.0655 |
| guaB3 | 0.0672 | 0.0407 | 0.0479 | 0.066 |
| lppI | 0.0574 | 0.0234 | 0.0834 | 0.059 |
| ctpF | 0.1037 | 0.0225 | 0.2564 | 0.1129 |
| pcnA | 0.0725 | 0.0257 | 0.1865 | 0.0794 |
| narK1 | 0.0741 | 0.0112 | 0.2074 | 0.0821 |
| glpD1 | 0.0487 | 0.0147 | 0.1252 | 0.0533 |
| fecB | 0.0796 | 0.0257 | 0.0761 | 0.0793 |
| plcC | 0.0913 | 0.0363 | 0.2282 | 0.0996 |
| narJ | 0.1095 | 0.0557 | 0.0552 | 0.1062 |
| fadB | 0.0664 | 0.0093 | 0.2074 | 0.0749 |
| purH | 0.434 | 0.4016 | 0.0834 | 0.4127 |
| glyA2 | 0.052 | 0.009 | 0.1804 | 0.0598 |
| lprL | 0.0249 | 0.0057 | 0.0761 | 0.028 |
| fusA1 | 0.1217 | 0.088 | 0.0834 | 0.1194 |
| thiO | 0.0945 | 0.0557 | 0.0761 | 0.0934 |
| mbtJ | 0.2985 | 0.1506 | 0.0761 | 0.2849 |
| parB | 0.0431 | 0.009 | 0.1178 | 0.0476 |
| lpdA | 0.0697 | 0.0277 | 0.0969 | 0.0713 |
| deaD | 0.049 | 0.0101 | 0.1656 | 0.056 |
| fbiC | 0.0526 | 0.0071 | 0.1387 | 0.0578 |
| espJ | 0.0651 | 0.023 | 0.146 | 0.07 |
| canA | 0.1731 | 0.1636 | 0.027 | 0.1642 |
| ugpA | 0.0624 | 0.0284 | 0.0687 | 0.0628 |
| sigF | 0.0574 | 0.0234 | 0.0834 | 0.059 |
| uvrC | 0.1237 | 0.0857 | 0.0896 | 0.1216 |
| espG1 | 0.0709 | 0.0182 | 0.1043 | 0.0729 |
| tgs4 | 0.0647 | 0.0157 | 0.1595 | 0.0704 |
| dapC | 0.0412 | 0.0128 | 0.1387 | 0.0471 |
| relA | 0.2209 | 0.1156 | 0.0969 | 0.2133 |
| moeB1 | 0.0896 | 0.0597 | 0.0969 | 0.09 |
| eccD5 | 0.0924 | 0.0271 | 0.1387 | 0.0952 |
| metA | 0.1171 | 0.0741 | 0.0834 | 0.115 |
| trcS | 0.1103 | 0.04 | 0.1521 | 0.1128 |
| lipW | 0.0572 | 0.0107 | 0.1595 | 0.0634 |
| valS | 0.0506 | 0.0088 | 0.1534 | 0.0568 |
| accD3 | 0.0846 | 0.0357 | 0.0761 | 0.084 |
| glnA1 | 0.0482 | 0.0234 | 0.0834 | 0.0503 |
| carA | 0.0987 | 0.028 | 0.0834 | 0.0977 |
| mmpL12 | 0.0784 | 0.0138 | 0.2908 | 0.0912 |
| vapC21 | 0.1237 | 0.0985 | 0.0417 | 0.1187 |
| ribF | 0.3284 | 0.2846 | 0.0687 | 0.3126 |
| mbtE | 0.1178 | 0.0352 | 0.2564 | 0.1261 |
| lppF | 0.0929 | 0.0424 | 0.1178 | 0.0944 |
| ureG | 0.0398 | 0.0157 | 0.0761 | 0.042 |
| htdZ | 0.0497 | 0.029 | 0.0552 | 0.05 |
| dnaZX | 0.0448 | 0.0182 | 0.1043 | 0.0484 |
| malQ | 0.0728 | 0.0126 | 0.2147 | 0.0814 |
| echA12 | 0.0398 | 0.0207 | 0.0761 | 0.042 |
| lipF | 0.199 | 0.0557 | 0.1178 | 0.194 |
| nirB | 0.0923 | 0.0284 | 0.2982 | 0.1047 |
| cyp132 | 0.0841 | 0.0175 | 0.146 | 0.0878 |
| zwf2 | 0.1076 | 0.0284 | 0.2221 | 0.1145 |
| ctpI | 0.0733 | 0.0051 | 0.4785 | 0.0978 |
| ceoC | 0.1015 | 0.0797 | 0.0626 | 0.0991 |
| cyp139 | 0.0373 | 0.0107 | 0.1043 | 0.0413 |
| ilvD | 0.0299 | 0.002 | 0.1252 | 0.0357 |
| accE5 | 0.3284 | 0.2355 | 0.0344 | 0.3105 |
| mpt70 | 0.0896 | 0.0757 | 0.0344 | 0.0862 |
| glgE | 0.0584 | 0.0114 | 0.1534 | 0.0641 |
| hemN | 0.0464 | 0.0157 | 0.1178 | 0.0507 |
| gcvB | 0.0846 | 0.0457 | 0.0761 | 0.0841 |
| fadD32 | 0.0299 | 0.0049 | 0.0834 | 0.0331 |
| lpqE | 0.0763 | 0.0357 | 0.0552 | 0.075 |
| eccD3 | 0.0384 | 0.01 | 0.1387 | 0.0445 |
| aftD | 0.0882 | 0.0121 | 0.2982 | 0.1009 |
| mce2D | 0.0653 | 0.0107 | 0.2147 | 0.0743 |
| aldC | 0.056 | 0.0182 | 0.1043 | 0.0589 |
| hisB | 0.0299 | 0.009 | 0.0552 | 0.0314 |
| fadA4 | 0.064 | 0.0128 | 0.1387 | 0.0685 |
| infB | 0.0459 | 0.0049 | 0.1742 | 0.0537 |
| lsr2 | 0.2687 | 0.3555 | 0 | 0.2525 |
| vapC12 | 0.1254 | 0.1156 | 0.027 | 0.1194 |
| drrA | 0.0299 | 0.0077 | 0.0626 | 0.0319 |
| gca | 0.0766 | 0.0218 | 0.1534 | 0.0812 |
| priA | 0.097 | 0.0332 | 0.1043 | 0.0974 |
| mce3F | 0.0393 | 0.0115 | 0.1252 | 0.0445 |
| ctpV | 0.0821 | 0.0122 | 0.2699 | 0.0934 |
| cysK2 | 0.0725 | 0.03 | 0.0896 | 0.0735 |
| agpS | 0.0439 | 0.0169 | 0.1104 | 0.0479 |
| ctaD | 0.2328 | 0.1756 | 0.0626 | 0.2224 |
| vapC43 | 0.0213 | 0.0128 | 0.0417 | 0.0225 |
| lysS | 0.0431 | 0.0224 | 0.0552 | 0.0438 |
| rslA | 0.057 | 0.023 | 0.0687 | 0.0577 |
| mce3D | 0.0584 | 0.0114 | 0.1521 | 0.064 |
| xerC | 0.1438 | 0.072 | 0.0687 | 0.1392 |
| leuA | 0.0564 | 0.0224 | 0.1178 | 0.0601 |
| tcrA | 0.0516 | 0.0284 | 0.0687 | 0.0526 |
| hpt | 0.0418 | 0.0197 | 0.0626 | 0.043 |
| gmhA | 0.0544 | 0.0169 | 0.1104 | 0.0578 |
| citE | 0.0597 | 0.0214 | 0.0896 | 0.0615 |
| fadE25 | 0.2361 | 0.1592 | 0.0687 | 0.2259 |
| gpdA1 | 0.1045 | 0.0482 | 0.1043 | 0.1044 |
| obg | 0.0801 | 0.0462 | 0.1252 | 0.0828 |
| fadE24 | 0.1924 | 0.0723 | 0.1178 | 0.1878 |
| moxR2 | 0.081 | 0.0642 | 0.0417 | 0.0786 |
| cysA1 | 0.0657 | 0.0317 | 0.0626 | 0.0655 |
| mce1B | 0.1282 | 0.0592 | 0.1104 | 0.1271 |
| devB | 0.203 | 0.1036 | 0.0626 | 0.1944 |
| zwf1 | 0.0651 | 0.0175 | 0.146 | 0.07 |
| grpE | 0.1294 | 0.0557 | 0.0344 | 0.1236 |
| pbpB | 0.0336 | 0.007 | 0.1043 | 0.0379 |
| glpD2 | 0.0962 | 0.049 | 0.0552 | 0.0937 |
| impA | 0.0512 | 0.0171 | 0.0896 | 0.0535 |
| kdpC | 0.0554 | 0.0471 | 0.0417 | 0.0546 |
| pepE | 0.0487 | 0.0115 | 0.1252 | 0.0533 |
| lppD | 0.033 | 0.0083 | 0.1252 | 0.0386 |
| murX | 0.0358 | 0.0137 | 0.0626 | 0.0374 |
| moaC1 | 0.2448 | 0.1456 | 0.0626 | 0.2337 |
| pks2 | 0.0845 | 0.0134 | 0.6528 | 0.1189 |
| rimJ | 0.1045 | 0.0782 | 0.0479 | 0.101 |
| lprG | 0.0697 | 0.0557 | 0.0344 | 0.0675 |
| fabH | 0.1244 | 0.0607 | 0.0761 | 0.1214 |
| nadR | 0.0398 | 0.0057 | 0.1595 | 0.047 |
| treX | 0.1114 | 0.0557 | 0.2012 | 0.1168 |
| pyrB | 0.0522 | 0.0332 | 0.0479 | 0.0519 |
| cfp29 | 0.0384 | 0.03 | 0.0417 | 0.0386 |
| argS | 0.1021 | 0.0399 | 0.1252 | 0.1035 |
| nuoJ | 0.0896 | 0.0857 | 0.0209 | 0.0854 |
| mbtN | 0.0353 | 0.0175 | 0.0687 | 0.0373 |
| dhaA | 0.145 | 0.0771 | 0.0896 | 0.1416 |
| fadD25 | 0.0566 | 0.0081 | 0.1939 | 0.0649 |
| acrA1 | 0.0781 | 0.0059 | 0.319 | 0.0927 |
| pyrH | 0.0537 | 0.0197 | 0.0969 | 0.0563 |
| yrbE3B | 0.0378 | 0.0117 | 0.0969 | 0.0414 |
| rpsL | 1 | 1 | 0.0209 | 0.9406 |
| erg3 | 0.1317 | 0.0768 | 0.1104 | 0.1304 |
| mce1R | 0.0846 | 0.0607 | 0.0761 | 0.0841 |
| lspA | 0.0821 | 0.0407 | 0.0479 | 0.08 |
| lysX | 0.0739 | 0.0057 | 0.2847 | 0.0866 |
| lipC | 0.1015 | 0.0677 | 0.0969 | 0.1012 |
| xseA | 0.0736 | 0.0317 | 0.0969 | 0.075 |
| amt | 0.0418 | 0.0101 | 0.1669 | 0.0494 |
| gpdA2 | 0.1493 | 0.072 | 0.0687 | 0.1443 |
| echA16 | 0.0497 | 0.0197 | 0.0969 | 0.0525 |
| fadD23 | 0.1338 | 0.0623 | 0.1804 | 0.1366 |
| PE14 | 0.1095 | 0.1156 | 0.0135 | 0.1037 |
| pbpA | 0.0697 | 0.0224 | 0.1804 | 0.0764 |
| pgsA1 | 0.1612 | 0.1516 | 0.027 | 0.1531 |
| mca | 0.064 | 0.0471 | 0.0417 | 0.0626 |
| ldtB | 0.0299 | 0.0032 | 0.1043 | 0.0344 |
| nusB | 0.0697 | 0.0757 | 0.0135 | 0.0663 |
| pckA | 0.0564 | 0.009 | 0.1804 | 0.0639 |
| alkB | 0.1151 | 0.0557 | 0.0896 | 0.1135 |
| sigA | 0.0418 | 0.0197 | 0.0626 | 0.043 |
| fadD7 | 0.1082 | 0.0407 | 0.1043 | 0.1079 |
| mgtE | 0.0707 | 0.0147 | 0.1252 | 0.074 |
| fadD14 | 0.1134 | 0.0707 | 0.1313 | 0.1145 |
| trmU | 0.1365 | 0.0557 | 0.0896 | 0.1336 |
| manA | 0.0299 | 0.0182 | 0.0479 | 0.031 |
| dapE | 0.0448 | 0.0557 | 0.0209 | 0.0434 |
| echA10 | 0.0712 | 0.0465 | 0.0834 | 0.0719 |
| fadE23 | 0.2388 | 0.0942 | 0.0896 | 0.2296 |
| aftB | 0.0546 | 0.0081 | 0.1939 | 0.063 |
| rpfB | 0.194 | 0.1106 | 0.0761 | 0.1868 |
| echA9 | 0.0448 | 0.0182 | 0.1043 | 0.0484 |
| fadE4 | 0.0537 | 0.0077 | 0.2012 | 0.0626 |
| glnE | 0.079 | 0.0134 | 0.2282 | 0.088 |
| glgC | 0.0373 | 0.0107 | 0.1043 | 0.0413 |
| asnB | 0.0436 | 0.0049 | 0.173 | 0.0514 |
| ndh | 0.0431 | 0.0224 | 0.0552 | 0.0438 |
| papA2 | 0.1109 | 0.0343 | 0.1865 | 0.1154 |
| accD2 | 0.0487 | 0.0147 | 0.1252 | 0.0533 |
| pntB | 0.0436 | 0.0142 | 0.0834 | 0.046 |
| plcB | 0.0962 | 0.0268 | 0.1804 | 0.1013 |
| iniB | 0.0733 | 0.0339 | 0.146 | 0.0777 |
| murF | 0.0551 | 0.0096 | 0.173 | 0.0622 |
| amiA2 | 0.0746 | 0.0357 | 0.0761 | 0.0747 |
| hsdM | 0.0704 | 0.0221 | 0.1656 | 0.0761 |
| mmpL7 | 0.0547 | 0.0057 | 0.2429 | 0.0661 |
| eccD1 | 0.086 | 0.0098 | 0.2282 | 0.0946 |
| whiB6 | 0.1684 | 0.06 | 0.1865 | 0.1694 |
| mmaA1 | 0.0384 | 0.0214 | 0.0417 | 0.0386 |
| atsB | 0.1675 | 0.0606 | 0.3325 | 0.1774 |
| fgd2 | 0.099 | 0.0462 | 0.1252 | 0.1005 |
| metH | 0.0676 | 0.0147 | 0.389 | 0.0871 |
| ppk2 | 0.0522 | 0.0407 | 0.0479 | 0.0519 |
| ftsZ | 0.1066 | 0.0471 | 0.0417 | 0.1026 |
| tmk | 0.2015 | 0.1681 | 0.0479 | 0.1922 |
| hemB | 0.1263 | 0.0741 | 0.0834 | 0.1237 |
| kshA | 0.039 | 0.0142 | 0.0834 | 0.0417 |
| ubiA | 0.0995 | 0.0657 | 0.0761 | 0.0981 |
| mycP3 | 0.0925 | 0.0467 | 0.1313 | 0.0948 |
| treS | 0.0622 | 0.0282 | 0.1595 | 0.0681 |
| mmpL5 | 0.1095 | 0.028 | 0.2638 | 0.1188 |
| mmpL9 | 0.0771 | 0.0132 | 0.3264 | 0.0922 |
| hemA | 0.0378 | 0.0117 | 0.0969 | 0.0414 |
| PE17 | 0.1147 | 0.0525 | 0.1252 | 0.1153 |
| aroK | 0.0657 | 0.0317 | 0.0626 | 0.0655 |
| echA18 | 0.0647 | 0.0257 | 0.0761 | 0.0654 |
| glnH | 0.0597 | 0.0317 | 0.0626 | 0.0599 |
| hsdS | 0.039 | 0.0234 | 0.0834 | 0.0417 |
| echA3 | 0.203 | 0.1456 | 0.0626 | 0.1944 |
| ephD | 0.1995 | 0.1567 | 0.1252 | 0.195 |
| mesT | 0.0697 | 0.0357 | 0.0761 | 0.0701 |
| dedA | 0.1125 | 0.0741 | 0.0834 | 0.1107 |
| tlyA | 0.0746 | 0.0407 | 0.0479 | 0.073 |
| hab | 0.1045 | 0.0657 | 0.0761 | 0.1027 |
| yjcE | 0.0763 | 0.0224 | 0.1178 | 0.0788 |
| phyA | 0.2175 | 0.167 | 0.0454 | 0.207 |
| cyp141 | 0.0423 | 0.0082 | 0.1595 | 0.0494 |
| cyp140 | 0.0448 | 0.0077 | 0.1325 | 0.0501 |
| lipE | 0.0488 | 0.0066 | 0.146 | 0.0547 |
| htdY | 0.0617 | 0.0197 | 0.0969 | 0.0638 |
| fadE31 | 0.0299 | 0.0043 | 0.0896 | 0.0335 |
| moaD1 | 0.1731 | 0.1156 | 0.0307 | 0.1644 |
| mutT3 | 0.0955 | 0.0557 | 0.0626 | 0.0935 |
| speE | 0.1301 | 0.0389 | 0.1656 | 0.1322 |
| TB8.4 | 0.0597 | 0.0457 | 0.0344 | 0.0582 |
| cycA | 0.0461 | 0.0121 | 0.146 | 0.0521 |
| mesJ | 0.0418 | 0.0437 | 0.0294 | 0.041 |
| mce4C | 0.0597 | 0.0257 | 0.0761 | 0.0607 |
| mshB | 0.081 | 0.0471 | 0.0417 | 0.0786 |
| choD | 0.0459 | 0.0072 | 0.173 | 0.0536 |
| pknH | 0.1061 | 0.0457 | 0.2429 | 0.1144 |
| mce1F | 0.0634 | 0.007 | 0.2147 | 0.0725 |
| fbpB | 0.086 | 0.0451 | 0.1104 | 0.0874 |
| lppV | 0.0678 | 0.0284 | 0.0687 | 0.0678 |
| sugA | 0.064 | 0.0471 | 0.0417 | 0.0626 |
| murI | 0.0537 | 0.0317 | 0.0626 | 0.0542 |
| acg | 0.0896 | 0.0465 | 0.0834 | 0.0892 |
| mdh | 0.191 | 0.1336 | 0.0626 | 0.1832 |
| lhr | 0.0663 | 0.0079 | 0.4025 | 0.0867 |
| fadD24 | 0.0299 | 0 | 0.1865 | 0.0394 |
| moxR1 | 0.0398 | 0.0157 | 0.0761 | 0.042 |
| fadD10 | 0.0299 | 0.0024 | 0.1178 | 0.0352 |
| lipR | 0.1927 | 0.1047 | 0.0687 | 0.1851 |
| fadE21 | 0.0537 | 0.0197 | 0.0969 | 0.0563 |
| gshA | 0.0528 | 0.0188 | 0.0834 | 0.0546 |
| mez | 0.0827 | 0.0142 | 0.1742 | 0.0882 |
| hemL | 0.3881 | 0.3555 | 0.0417 | 0.3671 |
| mapA | 0.1443 | 0.0857 | 0.0761 | 0.1401 |
| cobIJ | 0.061 | 0.014 | 0.1521 | 0.0665 |
| glnB | 0.0149 | 0.0257 | 0.0209 | 0.0153 |
| dapA | 0.1692 | 0.1056 | 0.0761 | 0.1635 |
| aroB | 0.1174 | 0.0677 | 0.0969 | 0.1161 |
| ctpD | 0.0431 | 0.0157 | 0.1178 | 0.0476 |
| ppnK | 0.2421 | 0.089 | 0.0552 | 0.2306 |
| glnA3 | 0.0299 | 0.002 | 0.1252 | 0.0357 |
| fadE30 | 0.0666 | 0.0418 | 0.0834 | 0.0676 |
| glcB | 0.105 | 0.0623 | 0.1804 | 0.1095 |
| narI | 0.0955 | 0.0557 | 0.0626 | 0.0935 |
| mntH | 0.197 | 0.1216 | 0.0626 | 0.1888 |
| fgd1 | 0.1373 | 0.0956 | 0.0969 | 0.1348 |
| vapB43 | 0.5871 | 0.5553 | 0.0135 | 0.5523 |
| mhpE | 0.1773 | 0.0522 | 0.1104 | 0.1731 |
| csd | 0.0456 | 0.0083 | 0.1252 | 0.0504 |
| modA | 0.1642 | 0.1306 | 0.0479 | 0.1571 |
| lppU | 0.0697 | 0.0557 | 0.0344 | 0.0675 |
| leuB | 0.062 | 0.0234 | 0.0834 | 0.0633 |
| radA | 0.1055 | 0.0277 | 0.0969 | 0.1049 |
| pks4 | 0.0488 | 0.0045 | 0.2773 | 0.0626 |
| cyp124 | 0.1559 | 0.039 | 0.1178 | 0.1535 |
| lpqL | 0.081 | 0.0214 | 0.0896 | 0.0815 |
| eccA3 | 0.0485 | 0.0145 | 0.1043 | 0.0519 |
| dctA | 0.1418 | 0.1231 | 0.0479 | 0.1361 |
| moeA1 | 0.0725 | 0.0557 | 0.0417 | 0.0706 |
| lipT | 0.0814 | 0.0284 | 0.146 | 0.0853 |
| atpC | 0 | 0.0557 | 0.0074 | 0.0005 |
| yrbE1B | 0.0456 | 0.0083 | 0.1252 | 0.0504 |
| fadB4 | 0.1134 | 0.0677 | 0.0626 | 0.1103 |
| fadD30 | 0.1327 | 0.034 | 0.319 | 0.1439 |
| espK | 0.0497 | 0.0124 | 0.2429 | 0.0614 |
| vapC31 | 0.1748 | 0.0985 | 0.0417 | 0.1667 |
| mmaA3 | 0.0512 | 0.0214 | 0.0896 | 0.0535 |
| fadE5 | 0.1612 | 0.0722 | 0.2699 | 0.1677 |
| sugI | 0.0597 | 0.0157 | 0.1178 | 0.0632 |
| rho | 0.0469 | 0.0214 | 0.0896 | 0.0495 |
| cyp123 | 0.1426 | 0.0623 | 0.1178 | 0.141 |
| amiD | 0.1537 | 0.0623 | 0.1804 | 0.1552 |
| vapC32 | 0.0896 | 0.0757 | 0.0344 | 0.0862 |
| ansP1 | 0.038 | 0.0093 | 0.146 | 0.0445 |
| hpx | 0.0344 | 0.0096 | 0.0834 | 0.0374 |
| eccB2 | 0.0522 | 0.0047 | 0.2712 | 0.0654 |
| sseB | 0.0816 | 0.0317 | 0.0969 | 0.0825 |
| mshA | 0.0776 | 0.0497 | 0.1313 | 0.0808 |
| ptpA | 0.0776 | 0.0677 | 0.027 | 0.0745 |
| leuS | 0.0602 | 0.0033 | 0.4307 | 0.0826 |
| mycP4 | 0.0624 | 0.0121 | 0.146 | 0.0674 |
| fbpA | 0.0896 | 0.0426 | 0.1521 | 0.0934 |
| sppA | 0.0497 | 0.0082 | 0.1595 | 0.0563 |
| kasA | 0.0497 | 0.0257 | 0.0761 | 0.0513 |
| cyp128 | 0.0709 | 0.0145 | 0.2147 | 0.0796 |
| mctB | 0.0537 | 0.0317 | 0.0626 | 0.0542 |
| nuoH | 0.0497 | 0.0257 | 0.0761 | 0.0513 |
| celA1 | 0.2529 | 0.1598 | 0.1252 | 0.2451 |
| fadD28 | 0.072 | 0.0169 | 0.1104 | 0.0743 |
| eno | 0.0378 | 0.0117 | 0.0969 | 0.0414 |
| uvrA | 0.1076 | 0.0375 | 0.2221 | 0.1145 |
| dnaE2 | 0.0911 | 0.0234 | 0.2638 | 0.1015 |
| ileS | 0.0805 | 0.0321 | 0.2221 | 0.0891 |
| clpB | 0.0361 | 0.0083 | 0.1252 | 0.0415 |
| lpqA | 0.1625 | 0.109 | 0.0552 | 0.1559 |
| gcpE | 0.1045 | 0.0707 | 0.0479 | 0.101 |
| serA2 | 0.0716 | 0.0317 | 0.0626 | 0.071 |
| accD4 | 0.0299 | 0.0057 | 0.0761 | 0.0327 |
| treY | 0.0517 | 0.0074 | 0.2773 | 0.0654 |
| lprD | 0.1592 | 0.1056 | 0.0344 | 0.1516 |
| pepA | 0.1028 | 0.069 | 0.0552 | 0.0999 |
| murD | 0.0833 | 0.0336 | 0.1252 | 0.0858 |
| dnaN | 0.0418 | 0.0117 | 0.2012 | 0.0514 |
| tgs1 | 0.1528 | 0.1015 | 0.1104 | 0.1502 |
| rpoA | 0.0725 | 0.0385 | 0.0896 | 0.0735 |
| mce1D | 0.0821 | 0.0307 | 0.1595 | 0.0868 |
| dxr | 0.1194 | 0.1006 | 0.0479 | 0.115 |
| ppsE | 0.0941 | 0.015 | 0.3607 | 0.1102 |
| folD | 0.0497 | 0.029 | 0.0552 | 0.05 |
| gmk | 0.1493 | 0.1396 | 0.027 | 0.1419 |
| cyp126 | 0.1457 | 0.1015 | 0.1104 | 0.1435 |
| blaI | 0.1418 | 0.1231 | 0.0479 | 0.1361 |
| embA | 0.0715 | 0.032 | 0.2908 | 0.0848 |
| mazF9 | 0.0896 | 0.0857 | 0.0209 | 0.0854 |
| cyp137 | 0.0409 | 0.0046 | 0.1804 | 0.0493 |
| lpqO | 0.0341 | 0.0086 | 0.0896 | 0.0374 |
| cyp143 | 0.209 | 0.163 | 0.1252 | 0.2039 |
| bioF2 | 0.0554 | 0.0086 | 0.3816 | 0.0752 |
| PE24 | 0.0418 | 0.0117 | 0.0969 | 0.0451 |
| pyrG | 0.1151 | 0.06 | 0.1865 | 0.1194 |
| carB | 0.0657 | 0.0107 | 0.2699 | 0.078 |
| qcrA | 0.0796 | 0.0557 | 0.0761 | 0.0794 |
| eccA1 | 0.0663 | 0.0224 | 0.1178 | 0.0694 |
| lppL | 0.062 | 0.0234 | 0.0834 | 0.0633 |
| rne | 0.0522 | 0.0145 | 0.2147 | 0.062 |
| ftsE | 0.0522 | 0.0332 | 0.0479 | 0.0519 |
| PE3 | 0.0443 | 0.0066 | 0.2221 | 0.0551 |
| glfT2 | 0.0613 | 0.021 | 0.1252 | 0.0651 |
| fbpD | 0.0597 | 0.0214 | 0.0896 | 0.0615 |
| pstC2 | 0.1559 | 0.0623 | 0.0552 | 0.1497 |
| eccE1 | 0.0574 | 0.0096 | 0.173 | 0.0644 |
| dlaT | 0.0497 | 0.0128 | 0.1387 | 0.0551 |
| recG | 0.1194 | 0.0775 | 0.146 | 0.121 |
| glgB | 0.0478 | 0.0077 | 0.1325 | 0.0529 |
| qcrC | 0.081 | 0.0642 | 0.0417 | 0.0786 |
| moeB2 | 0.1161 | 0.0623 | 0.0552 | 0.1124 |
| tgs3 | 0.0657 | 0.0557 | 0.027 | 0.0633 |
| fadE7 | 0.0464 | 0.0124 | 0.1178 | 0.0507 |
| lpqQ | 0.0567 | 0.0137 | 0.1313 | 0.0612 |
| vapB34 | 0 | 0.0557 | 0.0074 | 0.0005 |
| metK | 0.1194 | 0.0257 | 0.1325 | 0.1201 |
| espI | 0.1104 | 0.0197 | 0.2699 | 0.12 |
| lprE | 0.0776 | 0.0437 | 0.027 | 0.0745 |
| eccC4 | 0.0525 | 0.0029 | 0.3399 | 0.0699 |
| dnaE1 | 0.0657 | 0.0237 | 0.2012 | 0.0739 |
| cmaA1 | 0.0564 | 0.0357 | 0.0552 | 0.0563 |
| mycP2 | 0.0859 | 0.0103 | 0.2221 | 0.0941 |
| dppA | 0.0746 | 0.0307 | 0.1595 | 0.0797 |
| tesB1 | 0.1119 | 0.0782 | 0.0479 | 0.108 |
| PE1 | 0.0464 | 0.0057 | 0.2429 | 0.0583 |
| vapC6 | 0.2004 | 0.1156 | 0.0454 | 0.1909 |
| gabD2 | 0.1433 | 0.0647 | 0.1313 | 0.1425 |
| engA | 0.0516 | 0.0121 | 0.146 | 0.0573 |
| ilvX | 0.0839 | 0.0271 | 0.1387 | 0.0872 |
| viuB | 0.0682 | 0.0257 | 0.0896 | 0.0695 |
| atpG | 0.1433 | 0.0857 | 0.1313 | 0.1425 |
| moeW | 0.1104 | 0.0407 | 0.1313 | 0.1116 |
| ctpB | 0.0657 | 0.0297 | 0.2012 | 0.0739 |
| gyrA | 0.0516 | 0.0039 | 0.2982 | 0.0665 |
| vapC3 | 0.0896 | 0.0757 | 0.0344 | 0.0862 |
| accA2 | 0.0815 | 0.0184 | 0.2491 | 0.0916 |
| arfA | 0.0299 | 0.0137 | 0.0626 | 0.0319 |
| cysM | 0.1178 | 0.0399 | 0.1252 | 0.1182 |
| tatD | 0.0299 | 0.0037 | 0.0969 | 0.0339 |
| vapC2 | 0.0841 | 0.0502 | 0.0687 | 0.0831 |
| thrS | 0.1652 | 0.0996 | 0.0969 | 0.161 |
| PE34 | 0.1294 | 0.0757 | 0.0135 | 0.1223 |
| cysK1 | 0.0896 | 0.0632 | 0.1043 | 0.0905 |
| orn | 0.1493 | 0.1156 | 0.0417 | 0.1427 |
| sigD | 0.0896 | 0.0657 | 0.0761 | 0.0888 |
| hisD | 0.0746 | 0.0332 | 0.1043 | 0.0764 |
| amiC | 0.0707 | 0.0273 | 0.1252 | 0.074 |
| rpsN1 | 0.0448 | 0.0557 | 0.0209 | 0.0434 |
| mce1C | 0.0547 | 0.0082 | 0.1595 | 0.061 |
| truA | 0.0365 | 0.0157 | 0.0552 | 0.0376 |
| rskA | 0.0509 | 0.0169 | 0.1104 | 0.0545 |
| cobB | 0.0672 | 0.0332 | 0.1043 | 0.0694 |
| fadE10 | 0.0787 | 0.0175 | 0.2221 | 0.0874 |
| purL | 0.1176 | 0.0557 | 0.1104 | 0.1171 |
| fas | 0.0679 | 0.0016 | 0.6245 | 0.1016 |
| mmsA | 0.0827 | 0.0257 | 0.173 | 0.0881 |
| pheA | 0.0678 | 0.0339 | 0.0687 | 0.0678 |
| cstA | 0.0415 | 0.0024 | 0.2429 | 0.0537 |
| fadE33 | 0.0516 | 0.0284 | 0.0687 | 0.0526 |
| eccD4 | 0.038 | 0.0066 | 0.146 | 0.0445 |
| otsA | 0.0657 | 0.0557 | 0.027 | 0.0633 |
| ephE | 0.0981 | 0.0814 | 0.0417 | 0.0947 |
| pta | 0.0528 | 0.0096 | 0.1742 | 0.0601 |
| oxcA | 0.0876 | 0.0286 | 0.2074 | 0.0948 |
| mce2C | 0.0532 | 0.0114 | 0.1521 | 0.0592 |
| lppE | 0.209 | 0.1336 | 0.0626 | 0.2001 |
| mpt64 | 0.1493 | 0.089 | 0.0552 | 0.1435 |
| fusA2 | 0.0837 | 0.022 | 0.2773 | 0.0954 |
| hemD | 0.0933 | 0.0444 | 0.1043 | 0.0939 |
| sdhA | 0.1106 | 0.0522 | 0.1104 | 0.1105 |
| cobN | 0.0802 | 0.0086 | 0.4785 | 0.1043 |
| rfbE | 0.0597 | 0.0407 | 0.0479 | 0.059 |
| fadD26 | 0.1851 | 0.0857 | 0.0626 | 0.1776 |
| ppm1 | 0.1134 | 0.0497 | 0.1313 | 0.1144 |
| recD | 0.0746 | 0.0214 | 0.1865 | 0.0813 |
| dinP | 0.0981 | 0.0243 | 0.1387 | 0.1005 |
| cobK | 0.0528 | 0.0234 | 0.0834 | 0.0546 |
| gnd1 | 0.0461 | 0.0121 | 0.146 | 0.0521 |
| fadE32 | 0.0697 | 0.0357 | 0.0761 | 0.0701 |
| pknK | 0.0884 | 0.0228 | 0.3472 | 0.104 |
| phoR | 0.0629 | 0.0021 | 0.3816 | 0.0822 |
| dinG | 0.0401 | 0.004 | 0.1939 | 0.0494 |
| lprQ | 0.041 | 0.0107 | 0.1043 | 0.0448 |
| moaA1 | 0.0682 | 0.0086 | 0.1865 | 0.0753 |
| espG2 | 0.0434 | 0.0066 | 0.146 | 0.0496 |
| tuf | 0.0776 | 0.0677 | 0.027 | 0.0745 |
| uvrD1 | 0.0561 | 0.0101 | 0.1656 | 0.0627 |
| dnaB | 0.0728 | 0.0293 | 0.1656 | 0.0784 |
| echA14 | 0.209 | 0.1423 | 0.0552 | 0.1996 |
| glyS | 0.0981 | 0.0814 | 0.0417 | 0.0947 |
| ansP2 | 0.0299 | 0.002 | 0.1252 | 0.0357 |
| argC | 0.0299 | 0.0043 | 0.0896 | 0.0335 |
| ggtB | 0.0376 | 0.0061 | 0.1521 | 0.0445 |
| umaA | 0.1521 | 0.0757 | 0.1387 | 0.1512 |
| lat | 0.0341 | 0.0043 | 0.1865 | 0.0433 |
| lpqW | 0.1071 | 0.031 | 0.1104 | 0.1072 |
| phoH1 | 0.1254 | 0.0437 | 0.1313 | 0.1257 |
| fxsA | 0.1194 | 0.1056 | 0.0344 | 0.1142 |
| lpqP | 0.1023 | 0.0257 | 0.0896 | 0.1015 |
| metC | 0.0469 | 0.0214 | 0.0896 | 0.0495 |
| TB16.3 | 0.1612 | 0.1636 | 0.027 | 0.1531 |
| mas | 0.0658 | 0.0023 | 0.4994 | 0.0921 |
| nrdZ | 0.0985 | 0.0302 | 0.2699 | 0.1088 |
| yajC | 0.0995 | 0.0857 | 0.0344 | 0.0955 |
| smc | 0.0789 | 0.0104 | 0.3055 | 0.0926 |
| cyp136 | 0.0829 | 0.0357 | 0.1178 | 0.085 |
| secG | 0.1493 | 0.1756 | 0.0061 | 0.1406 |
| ansA | 0.0424 | 0.0083 | 0.1252 | 0.0474 |
| egtE | 0.0393 | 0.0083 | 0.1252 | 0.0445 |
| sigC | 0.0896 | 0.0857 | 0.0209 | 0.0854 |
| fhaA | 0.0674 | 0.018 | 0.2356 | 0.0776 |
| dppD | 0.0574 | 0.0142 | 0.173 | 0.0644 |
| scoB | 0.0725 | 0.0257 | 0.0896 | 0.0735 |
| fadD8 | 0.0785 | 0.0179 | 0.1804 | 0.0846 |
| pncB1 | 0.2457 | 0.1341 | 0.0834 | 0.2358 |
| echA21 | 0.153 | 0.1081 | 0.1043 | 0.15 |
| gyrB | 0.1049 | 0.0311 | 0.2638 | 0.1145 |
| stp | 0.0402 | 0.0061 | 0.1521 | 0.047 |
| fadD11 | 0.0526 | 0.0214 | 0.1387 | 0.0578 |
| fpg | 0.0537 | 0.0277 | 0.0969 | 0.0563 |
| rpsQ | 0.1791 | 0.1306 | 0.0479 | 0.1711 |
| glbO | 0.2023 | 0.1623 | 0.0552 | 0.1933 |
| ribC | 0.1254 | 0.0917 | 0.027 | 0.1194 |
| thiC | 0.0244 | 0.0066 | 0.0687 | 0.0271 |
| gpm1 | 0.0725 | 0.0557 | 0.0417 | 0.0706 |
| uvrB | 0.0497 | 0.0157 | 0.0969 | 0.0525 |
| fadD34 | 0.1783 | 0.0418 | 0.2638 | 0.1834 |
| glpQ2 | 0.0746 | 0.0332 | 0.1043 | 0.0764 |
| topA | 0.0896 | 0.0377 | 0.1313 | 0.0921 |
| PE6 | 0.0358 | 0.0137 | 0.0626 | 0.0374 |
| pstB | 0.0736 | 0.0397 | 0.0969 | 0.075 |
| ctpE | 0.0579 | 0.0098 | 0.2282 | 0.0682 |
| ponA2 | 0.0341 | 0.0043 | 0.1865 | 0.0433 |
| papA1 | 0.0644 | 0.0115 | 0.2564 | 0.076 |
| yrbE4B | 0.0657 | 0.0557 | 0.027 | 0.0633 |
| nrdB | 0.0338 | 0.0077 | 0.0969 | 0.0376 |
| fadE22 | 0.0853 | 0.0343 | 0.1877 | 0.0915 |
| accA1 | 0.0528 | 0.0072 | 0.173 | 0.0601 |
| rpsA | 0.194 | 0.1456 | 0.0761 | 0.1868 |
| vapB44 | 0.0497 | 0.0557 | 0.0135 | 0.0475 |
| aldA | 0.0402 | 0.0061 | 0.1521 | 0.047 |
| PE27 | 0.0833 | 0.0367 | 0.1252 | 0.0858 |
| rnpA | 0.1045 | 0.0457 | 0.0761 | 0.1027 |
| fadD31 | 0.0526 | 0.0128 | 0.1387 | 0.0578 |
| trpD | 0.0896 | 0.0443 | 0.1387 | 0.0925 |
| rocE | 0.2406 | 0.1015 | 0.1104 | 0.2326 |
| fdxB | 0.1194 | 0.0819 | 0.1043 | 0.1185 |
| nrp | 0.0785 | 0.0068 | 0.6319 | 0.112 |
| fadE29 | 0.0746 | 0.0407 | 0.0761 | 0.0747 |
| cpsY | 0.0627 | 0.0137 | 0.1313 | 0.0668 |
| ctpH | 0.0855 | 0.0242 | 0.4025 | 0.1047 |
| frdA | 0.1125 | 0.0349 | 0.173 | 0.1161 |
| atpF | 0.0418 | 0.0437 | 0.027 | 0.0409 |
| cinA | 0.0987 | 0.0372 | 0.173 | 0.1032 |
| lppY | 0.0856 | 0.0437 | 0.0969 | 0.0863 |
| lppZ | 0.0564 | 0.0124 | 0.1178 | 0.0601 |
| sdaA | 0.0706 | 0.0311 | 0.146 | 0.0751 |
| arcA | 0.0431 | 0.0124 | 0.1178 | 0.0476 |
| ctpA | 0.1012 | 0.0396 | 0.2773 | 0.1118 |
| sigI | 0.1214 | 0.0517 | 0.0969 | 0.1199 |
| deoC | 0.0482 | 0.0142 | 0.0834 | 0.0503 |
| furA | 0.1716 | 0.1306 | 0.0479 | 0.1641 |
| gabP | 0.153 | 0.0707 | 0.1043 | 0.15 |
| ephA | 0.0509 | 0.0169 | 0.1104 | 0.0545 |
| recB | 0.092 | 0.0202 | 0.3325 | 0.1065 |
| hycP | 0.2338 | 0.0807 | 0.0761 | 0.2241 |
| mmpL4 | 0.0746 | 0.0163 | 0.2147 | 0.0831 |
| mutB | 0.0407 | 0.0066 | 0.146 | 0.0471 |
| rnc | 0.1095 | 0.1156 | 0.0135 | 0.1037 |
| fadB3 | 0.157 | 0.0739 | 0.1521 | 0.1566 |
| fadE17 | 0.0426 | 0.0214 | 0.0896 | 0.0454 |
| amiB1 | 0.0856 | 0.0437 | 0.0969 | 0.0863 |
| ldtA | 0.2549 | 0.1341 | 0.0834 | 0.2444 |
| eccA2 | 0.1023 | 0.0343 | 0.1865 | 0.1074 |
| rplI | 0.1045 | 0.0707 | 0.0479 | 0.101 |
| psd | 0.1373 | 0.1156 | 0.027 | 0.1306 |
| efpA | 0.0995 | 0.0257 | 0.0761 | 0.098 |
| fadE1 | 0.097 | 0.0407 | 0.1043 | 0.0974 |
| pntAa | 0.0478 | 0.0257 | 0.0626 | 0.0487 |
| pepN | 0.0657 | 0.0157 | 0.2012 | 0.0739 |
| vapC16 | 0.197 | 0.1636 | 0.0626 | 0.1888 |
| opcA | 0.1015 | 0.0357 | 0.0969 | 0.1012 |
| PE31 | 0.0746 | 0.0707 | 0.0209 | 0.0713 |
| moaA2 | 0.1119 | 0.0332 | 0.1043 | 0.1114 |
| gadB | 0.0179 | 0.0077 | 0.0626 | 0.0206 |
| pks6 | 0.0706 | 0.008 | 0.6037 | 0.1029 |
| mbtF | 0.065 | 0.0134 | 0.2294 | 0.0749 |
| vapC34 | 0.0224 | 0.0257 | 0.0515 | 0.0242 |
| emrB | 0.1015 | 0.0365 | 0.1669 | 0.1054 |
| metS | 0.0404 | 0.0098 | 0.1104 | 0.0446 |
| dxs2 | 0.0796 | 0.0382 | 0.1595 | 0.0844 |
| menE | 0.2601 | 0.2098 | 0.0417 | 0.2468 |
| lppC | 0.095 | 0.0611 | 0.0687 | 0.0934 |
| dacB1 | 0.1824 | 0.1623 | 0.0552 | 0.1747 |
| fadD22 | 0.0461 | 0.003 | 0.2221 | 0.0567 |
| aftA | 0.0932 | 0.0284 | 0.2221 | 0.101 |
| cut3 | 0.0896 | 0.0557 | 0.0687 | 0.0883 |
| serB1 | 0.0199 | 0.0157 | 0.0344 | 0.0208 |
| adhB | 0.1891 | 0.1356 | 0.0552 | 0.1809 |
| mscL | 0.1407 | 0.1242 | 0.0417 | 0.1347 |
| ald | 0.064 | 0.0257 | 0.0896 | 0.0655 |
| metE | 0.0995 | 0.0523 | 0.1178 | 0.1006 |
| ponA1 | 0.2192 | 0.1012 | 0.1939 | 0.2176 |
| mkl | 0.1171 | 0.0511 | 0.0834 | 0.115 |
| trxC | 0.1151 | 0.0642 | 0.0417 | 0.1106 |
| mmpL2 | 0.0787 | 0.0175 | 0.3742 | 0.0966 |
| ppsA | 0.0762 | 0.0065 | 0.4577 | 0.0993 |
| qcrB | 0.0522 | 0.0182 | 0.1043 | 0.0553 |
| htrA | 0.0896 | 0.0268 | 0.1804 | 0.0951 |
| eccC2 | 0.0709 | 0.0034 | 0.7497 | 0.112 |
| aspB | 0.1095 | 0.029 | 0.1178 | 0.1099 |
| mgtC | 0.0469 | 0.0214 | 0.0896 | 0.0495 |
| rfe | 0.0299 | 0.0107 | 0.0761 | 0.0327 |
| ppsD | 0.1074 | 0.0136 | 0.389 | 0.1244 |
| sucD | 0.0564 | 0.0357 | 0.0552 | 0.0563 |
| ndhA | 0.1532 | 0.0457 | 0.2012 | 0.156 |
| hflX | 0.0299 | 0.0028 | 0.1104 | 0.0348 |
| acpS | 0.3881 | 0.4754 | 0 | 0.3646 |
| fadE8 | 0.1079 | 0.0649 | 0.173 | 0.1118 |
| omt | 0.1221 | 0.0775 | 0.0687 | 0.1188 |
| PE8 | 0.0666 | 0.0418 | 0.0834 | 0.0676 |
| iniC | 0.038 | 0.0066 | 0.146 | 0.0445 |
| sirA | 0.1001 | 0.0345 | 0.1104 | 0.1007 |
| alkA | 0.039 | 0.0049 | 0.173 | 0.0471 |
| apa | 0.0858 | 0.0369 | 0.1043 | 0.0869 |
| gltB | 0.0779 | 0.0114 | 0.3117 | 0.092 |
| pks13 | 0.0813 | 0.0102 | 0.3951 | 0.1003 |
| zmp1 | 0.0621 | 0.0184 | 0.2491 | 0.0734 |
| lppB | 0.0666 | 0.0234 | 0.0834 | 0.0676 |
| PE20 | 0.2388 | 0.2655 | 0.0074 | 0.2248 |
| fadE6 | 0.0433 | 0.0073 | 0.2074 | 0.0532 |
| mce4F | 0.0516 | 0.0121 | 0.146 | 0.0573 |
| trpE | 0.0776 | 0.0357 | 0.0969 | 0.0787 |
| rplA | 0.197 | 0.0976 | 0.0626 | 0.1888 |
| secD | 0.1674 | 0.0557 | 0.1521 | 0.1664 |
| ppiB | 0.1194 | 0.0707 | 0.0479 | 0.115 |
| arsC | 0.0632 | 0.0257 | 0.2282 | 0.0732 |
| deoD | 0.0962 | 0.0623 | 0.0552 | 0.0937 |
| glpK | 0.1343 | 0.1006 | 0.0761 | 0.1307 |
| cyp135B1 | 0.1134 | 0.0617 | 0.1313 | 0.1144 |
| pks7 | 0.062 | 0.0019 | 0.7288 | 0.1024 |
| adk | 0.1294 | 0.1156 | 0.0344 | 0.1236 |
| aceE | 0.0731 | 0.0267 | 0.1939 | 0.0804 |
| fprA | 0.0833 | 0.0399 | 0.1252 | 0.0858 |
| PE23 | 0.0697 | 0.0357 | 0.0969 | 0.0713 |
| ephB | 0.1221 | 0.0393 | 0.146 | 0.1235 |
| fadD5 | 0.0856 | 0.0257 | 0.2012 | 0.0926 |
| ribG | 0.1692 | 0.129 | 0.0552 | 0.1623 |
| dipZ | 0.0657 | 0.0221 | 0.1656 | 0.0717 |
| ltp1 | 0.0384 | 0.0128 | 0.0896 | 0.0415 |
| blaR | 0.1891 | 0.1556 | 0.0344 | 0.1797 |
| rip | 0.1075 | 0.0857 | 0.0626 | 0.1048 |
| treZ | 0.0664 | 0.0189 | 0.2074 | 0.0749 |
| fadD36 | 0.1764 | 0.1211 | 0.0687 | 0.1698 |
| lipL | 0.0776 | 0.0357 | 0.0969 | 0.0787 |
| idsB | 0.0787 | 0.0393 | 0.146 | 0.0828 |
| rplE | 0.209 | 0.1636 | 0.027 | 0.1979 |
| dnaQ | 0.186 | 0.0557 | 0.0834 | 0.1797 |
| murB | 0.0821 | 0.0482 | 0.0479 | 0.08 |
| oppA | 0.0407 | 0.0066 | 0.146 | 0.0471 |
| eccCa1 | 0.074 | 0.014 | 0.3117 | 0.0884 |
| plsB2 | 0.0393 | 0.0115 | 0.1252 | 0.0445 |
| mce2F | 0.0394 | 0.0101 | 0.1656 | 0.047 |
| sapM | 0.0547 | 0.0082 | 0.1595 | 0.061 |
| pks9 | 0.1063 | 0.0317 | 0.3399 | 0.1204 |
| glgP | 0.065 | 0.0098 | 0.2282 | 0.0749 |
| pknL | 0.0634 | 0.0182 | 0.1043 | 0.0658 |
| embR | 0.2156 | 0.1489 | 0.0552 | 0.2058 |
| cmr | 0.0995 | 0.0557 | 0.0344 | 0.0955 |
| trmD | 0.0597 | 0.0407 | 0.0479 | 0.059 |
| pncA | 0.1216 | 0.0279 | 0.2773 | 0.131 |
| dinF | 0.0554 | 0.0128 | 0.1387 | 0.0604 |
| vapB2 | 0.3134 | 0.3105 | 0.0209 | 0.2957 |
| esxH | 0.0179 | 0.0197 | 0.0294 | 0.0186 |
| pepB | 0.1674 | 0.1287 | 0.1521 | 0.1664 |
| bglS | 0.0682 | 0.0214 | 0.2847 | 0.0813 |
| nuoG | 0.0409 | 0.0046 | 0.1804 | 0.0493 |
| embC | 0.084 | 0.0145 | 0.2147 | 0.0919 |
| acn | 0.0355 | 0.0071 | 0.1387 | 0.0417 |
| wbbL2 | 0.0816 | 0.0357 | 0.0969 | 0.0825 |
| pdc | 0.0753 | 0.0357 | 0.1387 | 0.0791 |
| moaE1 | 0.2388 | 0.2355 | 0.0209 | 0.2256 |
| ppgK | 0.0927 | 0.0273 | 0.1252 | 0.0946 |
| mpa | 0.0299 | 0.0057 | 0.0761 | 0.0327 |
| guaA | 0.2303 | 0.1242 | 0.0896 | 0.2217 |
| prfB | 0.0426 | 0.0171 | 0.0896 | 0.0454 |
| amiB2 | 0.0448 | 0.0257 | 0.0761 | 0.0467 |
| rpmB1 | 0.0829 | 0.0357 | 0.0552 | 0.0812 |
| kmtR | 0.1866 | 0.1681 | 0.0479 | 0.1782 |
| dnaJ2 | 0.1373 | 0.0797 | 0.0626 | 0.1327 |
| fadD4 | 0.0725 | 0.0214 | 0.2356 | 0.0824 |
| phoP | 0.0597 | 0.0857 | 0.0074 | 0.0565 |
| nat | 0.1113 | 0.072 | 0.0687 | 0.1087 |
| pks1 | 0.0611 | 0.0079 | 0.5755 | 0.0923 |
| ugpC | 0.2644 | 0.1242 | 0.0896 | 0.2537 |
| fadE34 | 0.0576 | 0.0086 | 0.1865 | 0.0654 |
| alr | 0.0706 | 0.0257 | 0.146 | 0.0751 |
| fadA2 | 0.0355 | 0.0071 | 0.1387 | 0.0417 |
| fadE9 | 0.0758 | 0.0418 | 0.0834 | 0.0762 |
| recC | 0.0482 | 0.0034 | 0.2638 | 0.0612 |
| bkdB | 0.0253 | 0.0049 | 0.0834 | 0.0288 |
| plsB1 | 0.0413 | 0.0049 | 0.173 | 0.0493 |
| echA8 | 0.1095 | 0.0757 | 0.0344 | 0.1049 |
| adhA | 0.0931 | 0.0451 | 0.1104 | 0.0941 |
| atpA | 0.081 | 0.0557 | 0.0896 | 0.0815 |
| nuoN | 0.055 | 0.0147 | 0.1252 | 0.0592 |
| irtB | 0.0458 | 0.0117 | 0.0969 | 0.0489 |
| mazF2 | 0.1891 | 0.1556 | 0.0135 | 0.1784 |
| PE16 | 0.0299 | 0.0017 | 0.1313 | 0.036 |
| bioD | 0.2289 | 0.1956 | 0.0552 | 0.2183 |
| fabG4 | 0.0611 | 0.0128 | 0.1387 | 0.0658 |
| purK | 0.0232 | 0.009 | 0.0552 | 0.0251 |
| guaB2 | 0.095 | 0.072 | 0.0687 | 0.0934 |
| pth | 0.0776 | 0.0377 | 0.0626 | 0.0767 |
| mutT4 | 0.0458 | 0.0157 | 0.0969 | 0.0489 |
| vapC27 | 0.4104 | 0.2056 | 0.0479 | 0.3882 |
| udgA | 0.0497 | 0.0157 | 0.1178 | 0.0538 |
| helZ | 0.0624 | 0.0039 | 0.2982 | 0.0767 |
| lipM | 0.1254 | 0.0637 | 0.0969 | 0.1236 |
| mmpS3 | 0.0547 | 0.0257 | 0.0761 | 0.056 |
| mbtD | 0.0843 | 0.0134 | 0.2294 | 0.093 |
| glnQ | 0.1071 | 0.0592 | 0.1104 | 0.1073 |
| pknD | 0.1049 | 0.0351 | 0.2356 | 0.1128 |
| ephF | 0.0474 | 0.0098 | 0.1104 | 0.0512 |
| hrcA | 0.0564 | 0.0157 | 0.1178 | 0.0601 |
| narG | 0.0856 | 0.0297 | 0.2012 | 0.0926 |
| rpmB2 | 0.0384 | 0.03 | 0.0417 | 0.0386 |
| eccB1 | 0.1084 | 0.0115 | 0.2564 | 0.1173 |
| fhaB | 0.0373 | 0.0257 | 0.0479 | 0.0379 |
| tpx | 0.1443 | 0.1006 | 0.0761 | 0.1401 |
| mmpL8 | 0.0393 | 0.001 | 0.389 | 0.0605 |
| TB31.7 | 0.0821 | 0.0294 | 0.1043 | 0.0834 |
| tyrA | 0.0825 | 0.031 | 0.1104 | 0.0842 |
| mprB | 0.1015 | 0.0637 | 0.0969 | 0.1012 |
| guaB1 | 0.0324 | 0.0035 | 0.1521 | 0.0396 |
| msrB | 0.0373 | 0.0257 | 0.0479 | 0.0379 |
| purC | 0.0353 | 0.0121 | 0.0687 | 0.0373 |
| mbtB | 0.1151 | 0.0248 | 0.2368 | 0.1224 |
| cysS1 | 0.199 | 0.0956 | 0.0761 | 0.1915 |
| pknB | 0.1703 | 0.0398 | 0.2282 | 0.1737 |
| fadD29 | 0.0874 | 0.0171 | 0.1865 | 0.0934 |
| oppD | 0.1401 | 0.0649 | 0.0834 | 0.1366 |
| fadD1 | 0.0829 | 0.0268 | 0.1804 | 0.0888 |
| icd2 | 0.0652 | 0.0046 | 0.1804 | 0.0721 |
| ctpC | 0.0418 | 0.0077 | 0.1313 | 0.0472 |
| fadH | 0.0713 | 0.019 | 0.2429 | 0.0817 |
| gltS | 0.1453 | 0.0877 | 0.0969 | 0.1423 |
| hycQ | 0.0858 | 0.0519 | 0.1043 | 0.0869 |
| pgmA | 0.0923 | 0.0448 | 0.146 | 0.0955 |
| oxyS | 0.062 | 0.028 | 0.0834 | 0.0633 |
| cbs | 0.064 | 0.03 | 0.0896 | 0.0655 |
| yrbE2A | 0.1045 | 0.0707 | 0.0761 | 0.1028 |
| embB | 0.085 | 0.0188 | 0.3534 | 0.1012 |
| ppk1 | 0.0981 | 0.0557 | 0.0896 | 0.0976 |
| epiA | 0.2328 | 0.1876 | 0.0626 | 0.2224 |
| miaA | 0.2303 | 0.1242 | 0.0896 | 0.2217 |
| coaA | 0.0299 | 0.0157 | 0.0552 | 0.0314 |
| ftsH | 0.0506 | 0.0218 | 0.1521 | 0.0567 |
| lpqF | 0.061 | 0.0114 | 0.1521 | 0.0665 |
| secA1 | 0.0597 | 0.0096 | 0.173 | 0.0665 |
| udgB | 0.064 | 0.0471 | 0.0454 | 0.0629 |
| lppG | 0.0796 | 0.0457 | 0.0761 | 0.0794 |
| mutY | 0.0858 | 0.0369 | 0.1043 | 0.0869 |
| plsC | 0.0975 | 0.0517 | 0.0969 | 0.0974 |
| citA | 0.0398 | 0.009 | 0.1178 | 0.0445 |
| accD1 | 0.0497 | 0.0257 | 0.0761 | 0.0513 |
| eccA5 | 0.0373 | 0.0107 | 0.1043 | 0.0413 |
| fixB | 0.0597 | 0.0317 | 0.0626 | 0.0599 |
| pks12 | 0.0875 | 0.0032 | 1 | 0.1428 |
| kshB | 0.0597 | 0.0182 | 0.1043 | 0.0624 |
| katG | 0.0552 | 0.003 | 0.4515 | 0.0792 |
| hisC1 | 0.1079 | 0.0649 | 0.0834 | 0.1064 |
| zur | 0.0384 | 0.0128 | 0.0896 | 0.0415 |
| chaA | 0.1834 | 0.1156 | 0.0417 | 0.1748 |
| idsA1 | 0.0336 | 0.0107 | 0.1043 | 0.0379 |
| secF | 0.1058 | 0.0584 | 0.146 | 0.1082 |
| sirR | 0.0579 | 0.0169 | 0.1104 | 0.0611 |
| nuoF | 0.0554 | 0.0214 | 0.0896 | 0.0574 |
| cobS | 0.1716 | 0.1531 | 0.0479 | 0.1641 |
| pflA | 0.0583 | 0.0128 | 0.1387 | 0.0631 |
| dnaA | 0.0654 | 0.0086 | 0.2847 | 0.0787 |
| hisC2 | 0.0597 | 0.0182 | 0.1043 | 0.0624 |
| cyp142 | 0.0682 | 0.03 | 0.0896 | 0.0695 |
| narK2 | 0.1095 | 0.0597 | 0.0969 | 0.1087 |
| glmU | 0.1703 | 0.0768 | 0.1104 | 0.1666 |
| pknE | 0.0361 | 0.0083 | 0.1252 | 0.0415 |
| tam | 0.209 | 0.1756 | 0.0344 | 0.1984 |
| sthA | 0.1459 | 0.0823 | 0.1178 | 0.1441 |
| mqo | 0.0531 | 0.0124 | 0.1178 | 0.057 |
| narK3 | 0.0459 | 0.0119 | 0.173 | 0.0536 |
| glnA4 | 0.1004 | 0.0448 | 0.146 | 0.1031 |
| adh | 0.0507 | 0.0107 | 0.1313 | 0.0556 |
| mazF7 | 0.1134 | 0.1036 | 0.027 | 0.1082 |
| fadE2 | 0.2799 | 0.1943 | 0.1043 | 0.2692 |
| pstA1 | 0.0497 | 0.0357 | 0.0552 | 0.05 |
| ctpJ | 0.1004 | 0.0502 | 0.2221 | 0.1077 |
| lipI | 0.0597 | 0.0257 | 0.1043 | 0.0624 |
| ideR | 0.0418 | 0.0437 | 0.027 | 0.0409 |
| ligA | 0.0617 | 0.0277 | 0.0969 | 0.0638 |
| yrbE4A | 0.1194 | 0.0857 | 0.0209 | 0.1134 |
| pssA | 0.2687 | 0.1456 | 0.0479 | 0.2552 |
| ispE | 0.1194 | 0.0669 | 0.1043 | 0.1184 |
| dosT | 0.092 | 0.0207 | 0.1595 | 0.096 |
| vapC41 | 0.1433 | 0.0976 | 0.0626 | 0.1384 |
| ddlA | 0.0554 | 0.0471 | 0.0417 | 0.0546 |
| mce3R | 0.2289 | 0.1823 | 0.0552 | 0.2183 |
| folC | 0.1397 | 0.0629 | 0.1656 | 0.1412 |
| vapB33 | 0.1194 | 0.0857 | 0.0209 | 0.1134 |
| helY | 0.0492 | 0.0054 | 0.2491 | 0.0613 |
| fabD | 0.0981 | 0.0814 | 0.0417 | 0.0947 |
| mapB | 0.1028 | 0.0623 | 0.0552 | 0.0999 |
| esxF | 0.0482 | 0.0234 | 0.0834 | 0.0503 |
| cobO | 0.081 | 0.0471 | 0.0896 | 0.0815 |
| narU | 0.0456 | 0.0178 | 0.1252 | 0.0504 |
| pknI | 0.0583 | 0.0128 | 0.1387 | 0.0631 |
| atpH | 0.081 | 0.0186 | 0.1387 | 0.0845 |
| lpqI | 0.0544 | 0.0239 | 0.1104 | 0.0578 |
| accD6 | 0.4104 | 0.2206 | 0.0479 | 0.3883 |
| hycE | 0.061 | 0.0114 | 0.1534 | 0.0666 |
| iniA | 0.0466 | 0.0107 | 0.2147 | 0.0568 |
| hisS | 0.0763 | 0.0424 | 0.0552 | 0.075 |
| aceAa | 0.1791 | 0.0942 | 0.0896 | 0.1736 |
| ftsY | 0.095 | 0.0502 | 0.0687 | 0.0934 |
| prpC | 0.1393 | 0.0857 | 0.0761 | 0.1354 |
| ggtA | 0.1263 | 0.0649 | 0.1742 | 0.1292 |
| espE | 0.2198 | 0.1429 | 0.146 | 0.2153 |
| pks8 | 0.0694 | 0.002 | 0.6528 | 0.1047 |
| acs | 0.0564 | 0.009 | 0.1804 | 0.0639 |
| mutT1 | 0.0528 | 0.0234 | 0.0834 | 0.0546 |
| ltp4 | 0.0841 | 0.0393 | 0.0687 | 0.0831 |
| ilvB2 | 0.0622 | 0.0107 | 0.1595 | 0.0681 |
| ethA | 0.0866 | 0.0206 | 0.2773 | 0.0981 |
| echA2 | 0.0579 | 0.0169 | 0.1104 | 0.0611 |
| hsaF | 0.1294 | 0.0956 | 0.0344 | 0.1236 |
| ffh | 0.0624 | 0.0284 | 0.0687 | 0.0628 |
| lipD | 0.0851 | 0.0224 | 0.1804 | 0.0908 |
| glnD | 0.0734 | 0.0119 | 0.2491 | 0.084 |
| mce2A | 0.057 | 0.0121 | 0.146 | 0.0624 |
| vapB10 | 0.1791 | 0.0857 | 0.0344 | 0.1702 |
| eccC3 | 0.0868 | 0.0162 | 0.2982 | 0.0996 |
| greA | 0.0299 | 0.0557 | 0.0172 | 0.0291 |
| trpS | 0.0537 | 0.0437 | 0.0282 | 0.0521 |
| esxD | 0.0365 | 0.0224 | 0.0564 | 0.0377 |
| lipP | 0.1493 | 0.1756 | 0.0074 | 0.1407 |
| sodA | 0.0179 | 0.0197 | 0.0282 | 0.0185 |
| adoK | 0.0398 | 0.0357 | 0.0356 | 0.0395 |
| rpsK | 0.0299 | 0.0197 | 0.0282 | 0.0298 |
| hsaD | 0.0398 | 0.0257 | 0.0356 | 0.0395 |
| rpsN2 | 0.0179 | 0.0197 | 0.0282 | 0.0185 |
| mbtI | 0.0365 | 0.029 | 0.0564 | 0.0377 |
| vapB32 | 0.0299 | 0.1156 | 0.0049 | 0.0285 |
| gltA2 | 0.0299 | 0.0107 | 0.0491 | 0.031 |
| nrdH | 0.0299 | 0.1156 | 0.0049 | 0.0285 |
| hisE | 0.1015 | 0.0677 | 0.0282 | 0.097 |
| sigK | 0.01 | 0.0357 | 0.0135 | 0.0102 |
| nuoI | 0.01 | 0.0357 | 0.0135 | 0.0102 |
| ruvA | 0.0299 | 0.1156 | 0.0049 | 0.0285 |
| esxS | 0.01 | 0.0357 | 0.0135 | 0.0102 |
| lppP | 0.0166 | 0.009 | 0.0564 | 0.019 |
| lpqS | 0.0597 | 0.0557 | 0.0356 | 0.0582 |
| vapC10 | 0.0497 | 0.0357 | 0.0356 | 0.0488 |
| dop | 0.0373 | 0.0257 | 0.0491 | 0.038 |
| pimA | 0.0299 | 0.0107 | 0.0491 | 0.031 |
| dnaK | 0.0537 | 0.0437 | 0.0282 | 0.0521 |
| mazE4 | 0.0299 | 0.0357 | 0.0356 | 0.0303 |
| prfA | 0.0299 | 0.0197 | 0.0282 | 0.0298 |
| vapB24 | 0.2328 | 0.2116 | 0.0282 | 0.2204 |
| esxL | 0.0995 | 0.0657 | 0.0356 | 0.0956 |
| thrC | 0.1731 | 0.1156 | 0.0282 | 0.1643 |
| whiB7 | 0.0537 | 0.0437 | 0.0282 | 0.0521 |
| vapB40 | 0.2687 | 0.2655 | 0.0209 | 0.2537 |
| mazE1 | 0.0896 | 0.1156 | 0.0074 | 0.0846 |
| TB18.6 | 0 | 0.0257 | 0.0209 | 0.0013 |
| rplT | 0.0149 | 0.0257 | 0.0209 | 0.0153 |
| vapB41 | 0.1493 | 0.2355 | 0 | 0.1403 |
| esxQ | 0.0418 | 0.0317 | 0.0282 | 0.041 |
| esxI | 0.1045 | 0.1006 | 0.0209 | 0.0994 |
| tatB | 0.0896 | 0.1156 | 0.0074 | 0.0846 |
| TB7.3 | 0.0896 | 0.1156 | 0.0074 | 0.0846 |
| lexA | 0.0199 | 0.0157 | 0.0356 | 0.0208 |
| vapC38 | 0.0384 | 0.0214 | 0.0429 | 0.0387 |
| end | 0.0213 | 0.0128 | 0.0429 | 0.0226 |
| tatA | 0.0149 | 0.0257 | 0.0209 | 0.0153 |
| nuoA | 0.0497 | 0.0757 | 0.0135 | 0.0475 |
| rpiB | 0.006 | 0.0197 | 0.0282 | 0.0074 |
| relK | 0.1493 | 0.1756 | 0.0074 | 0.1407 |
| proS | 0.0115 | 0.0096 | 0.0859 | 0.016 |
| cobU | 0.0398 | 0.0357 | 0.0356 | 0.0395 |
| prrA | 0.1095 | 0.1156 | 0.0135 | 0.1037 |
| smtB | 0.2209 | 0.2116 | 0.0282 | 0.2092 |
| hisH | 0.0384 | 0.0214 | 0.0429 | 0.0387 |
| argR | 0.1294 | 0.1356 | 0.0135 | 0.1224 |
| esxW | 0.0597 | 0.0857 | 0.0074 | 0.0565 |

Supplementary Table 4, Detailed scores of the remaining genes identified in the analysis of Rifampicin-resistant, Isoniazid-resistant, and sensitive samples from the CRyPTIC dataset. This table provides insights into the gene alterations and their relevance across the phenotypic groups.


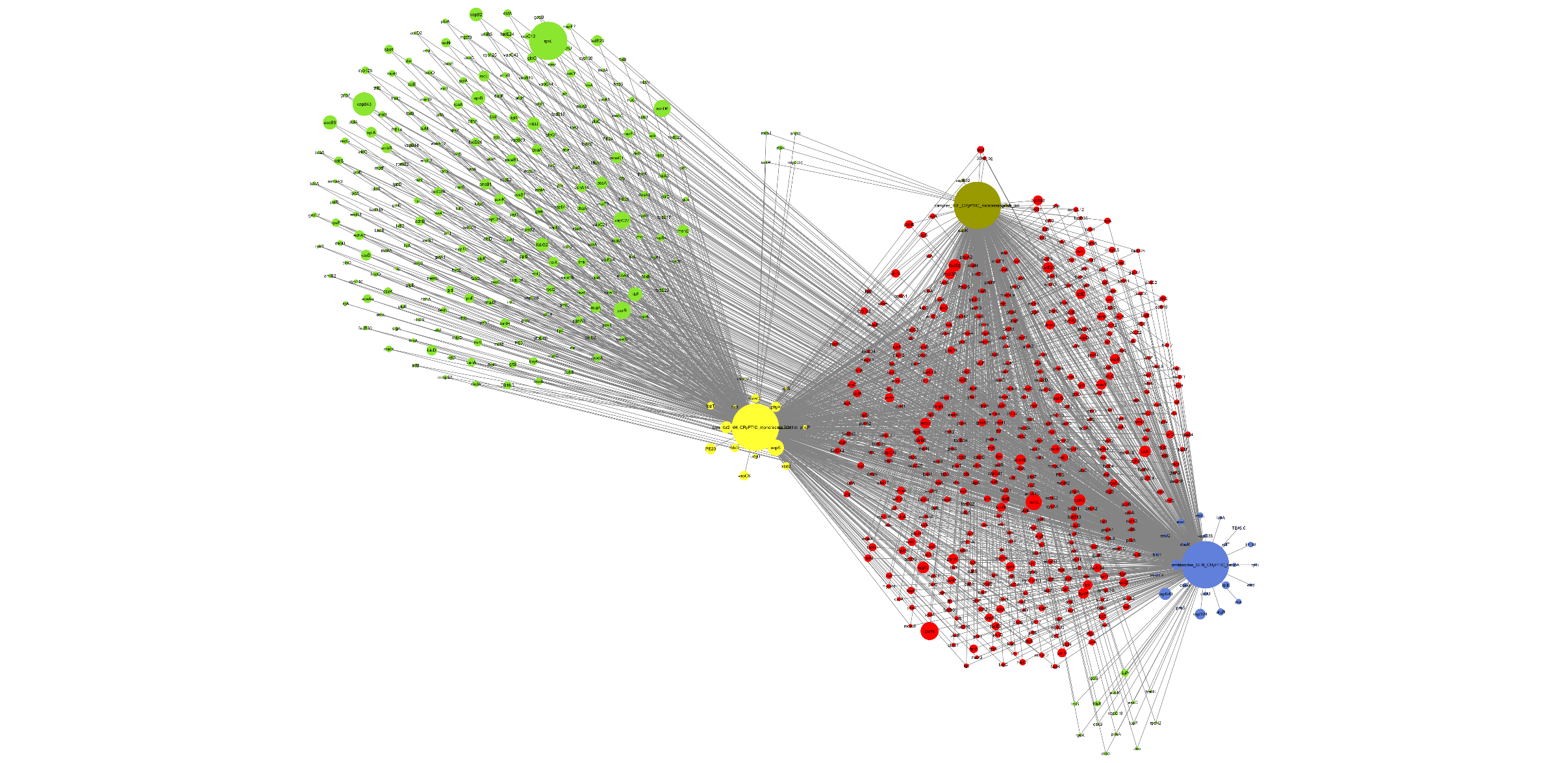


Supplementary figure 6, Final network of gene alterations in Rifampicin-resistant, Isoniazid-resistant, and sensitive Mycobacterium tuberculosis samples from the CRyPTIC dataset. This visualization captures the relationships between genes and phenotypic groups, with node size reflecting CAIS (gene impact) and edges denoting observed alterations. High-impact genes such as rpoB and katG are prominently linked to resistance phenotypes. The colour scheme employs three primary colours to represent genes exclusively associated with a single phenotype: red for Rifampicin-resistant, blue for Isoniazid-resistant, and green for Sensitive. Some gene nodes appear in distinct colours, indicating they harbor unique altered positions across multiple phenotypic groups. These were assigned separate colours to distinguish them from genes strictly linked to one phenotype, improving clarity and highlighting complex resistance patterns.

# **References**

| [1] | The CRyPTIC Consortium, "A data compendium associating the genomes of 12,289 Mycobacterium tuberculosis isolates with quantitative resistance phenotypes to 13 antibiotics," *PLoS biology,* vol. 20, no. 8, 2022. |
| --- | --- |
| [2] | M. T. Zaw, N. A. Emran and Z. Lin, "Mutations inside rifampicin-resistance determining region of rpoB gene associated with rifampicin-resistance in Mycobacterium tuberculosis.," *Journal of infection and public health,* vol. 11, no. 5, p. 605–610, 2018. |
| [3] | J. Cheng, L. Thibert, T. Sanchez, L. Heifets and Y. Zhang, "PncA Mutations as a Major Mechanism of Pyrazinamide Resistance in Mycobacterium tuberculosis: Spread of a Monoresistant Strain in Quebec, Canada," *Antimicrobial Agents and Chemotherapy,* vol. 44, no. 3, pp. 528-532, 2000. |
| [4] | P. Ma, T. Luo, L. Ge, Z. Chen, X. Wang, R. Zhao, W. Liao and L. Bao, "Compensatory effects of M. Tuberculosis rpoB mutations outside the rifampicin resistance-determining region," *Emerging Microbes & Infections,* vol. 10, no. 1, p. 743, 2021. |
| [5] | A. N. Unissa, N. Selvakumar, S. Narayanan, C. Suganthi and L. E. Hanna, "Investigation of Ser315 Substitutions within katG Gene in Isoniazid-Resistant Clinical Isolates of Mycobacterium tuberculosis from South India.," *BioMed Research International,* p. 257983, 2015. |
