# Supplementary figures and images for: FuNTB: a functional network clustering tool for the analysis of genome-wide genetic variants in Mycobacterium tuberculosis
Source: Bioinformatics. 2025 Jun 11;41(7):btaf341. doi: 10.1093/bioinformatics/btaf341 (PMC12255883; doi:10.1093/bioinformatics/btaf341)

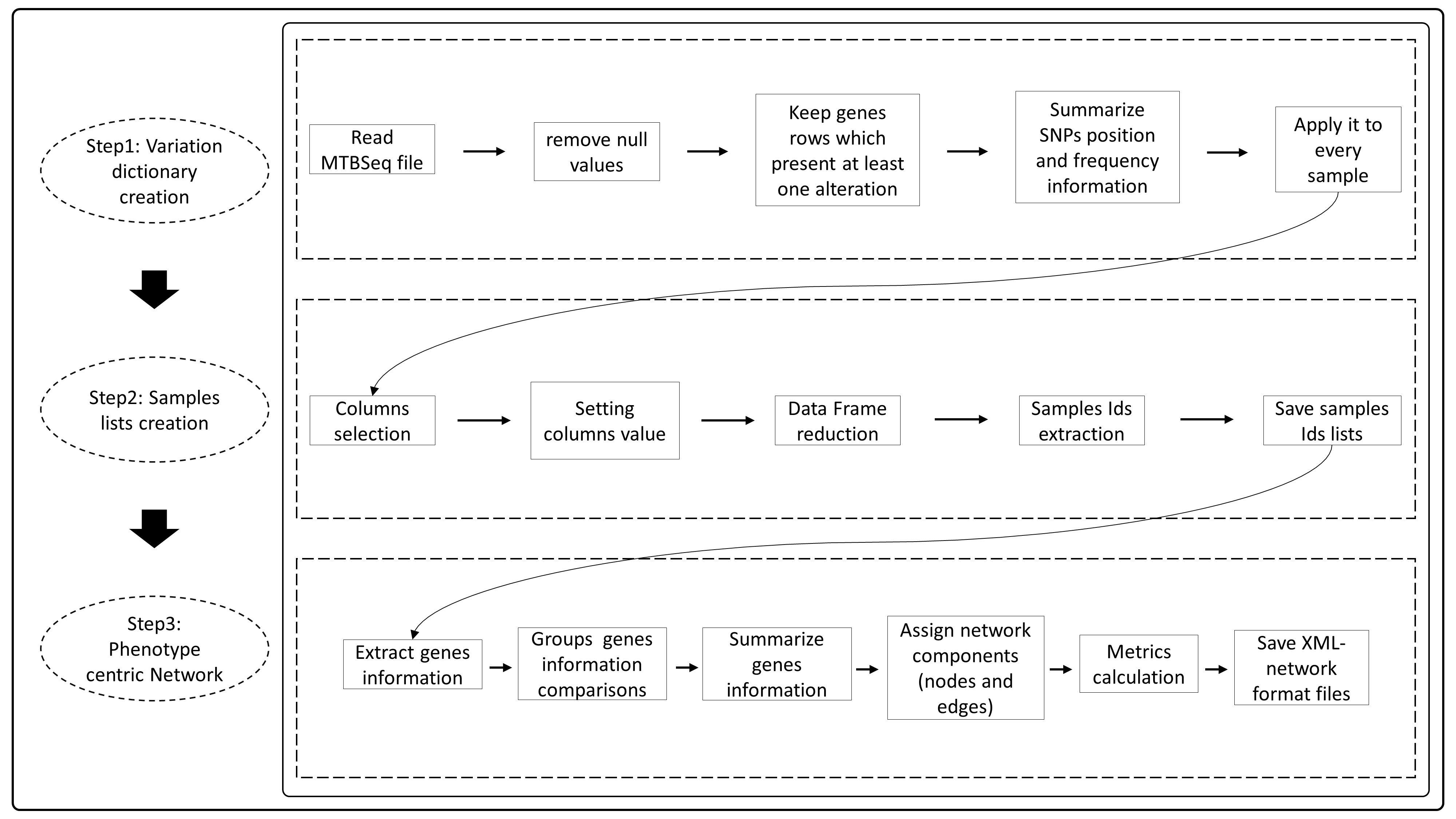

Supplement: btaf341_Supplementary_Data [file btaf341_supplementary_data.zip › Supplementary_figure_1.TIF]

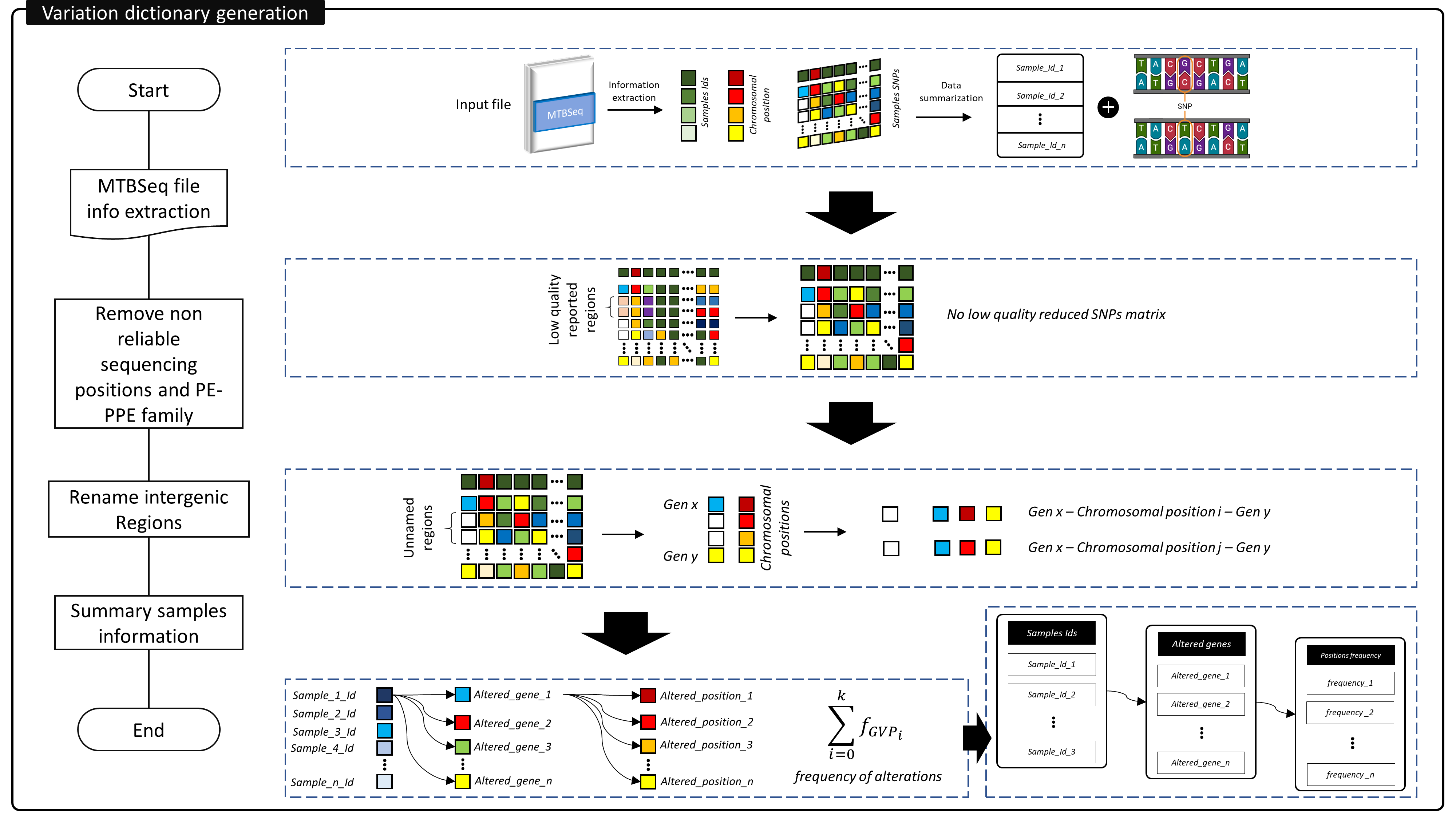

Supplement: btaf341_Supplementary_Data [file btaf341_supplementary_data.zip › Supplementary_Figure_2.TIF]

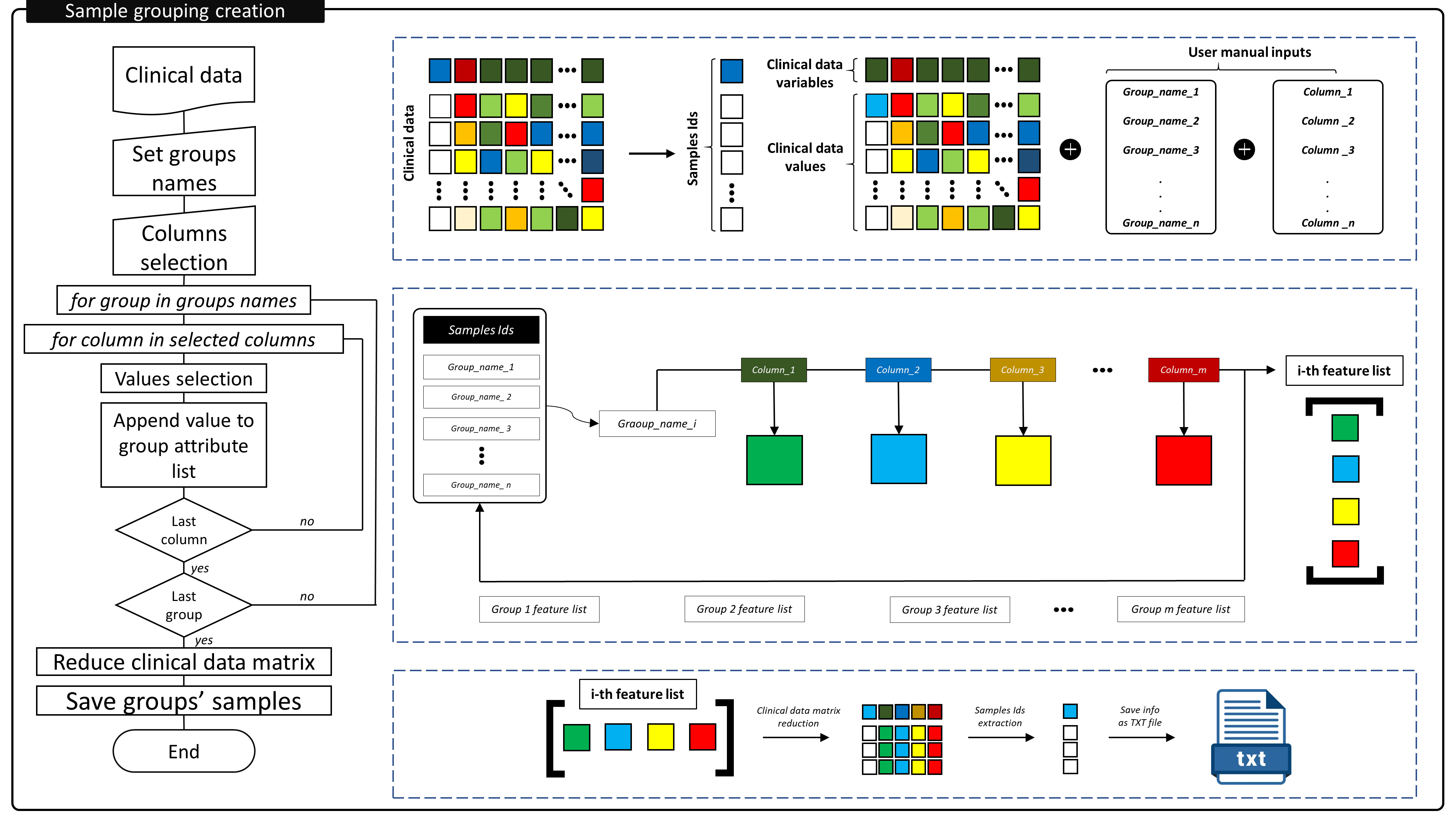

Supplement: btaf341_Supplementary_Data [file btaf341_supplementary_data.zip › Supplementary_Figure_3.TIF]

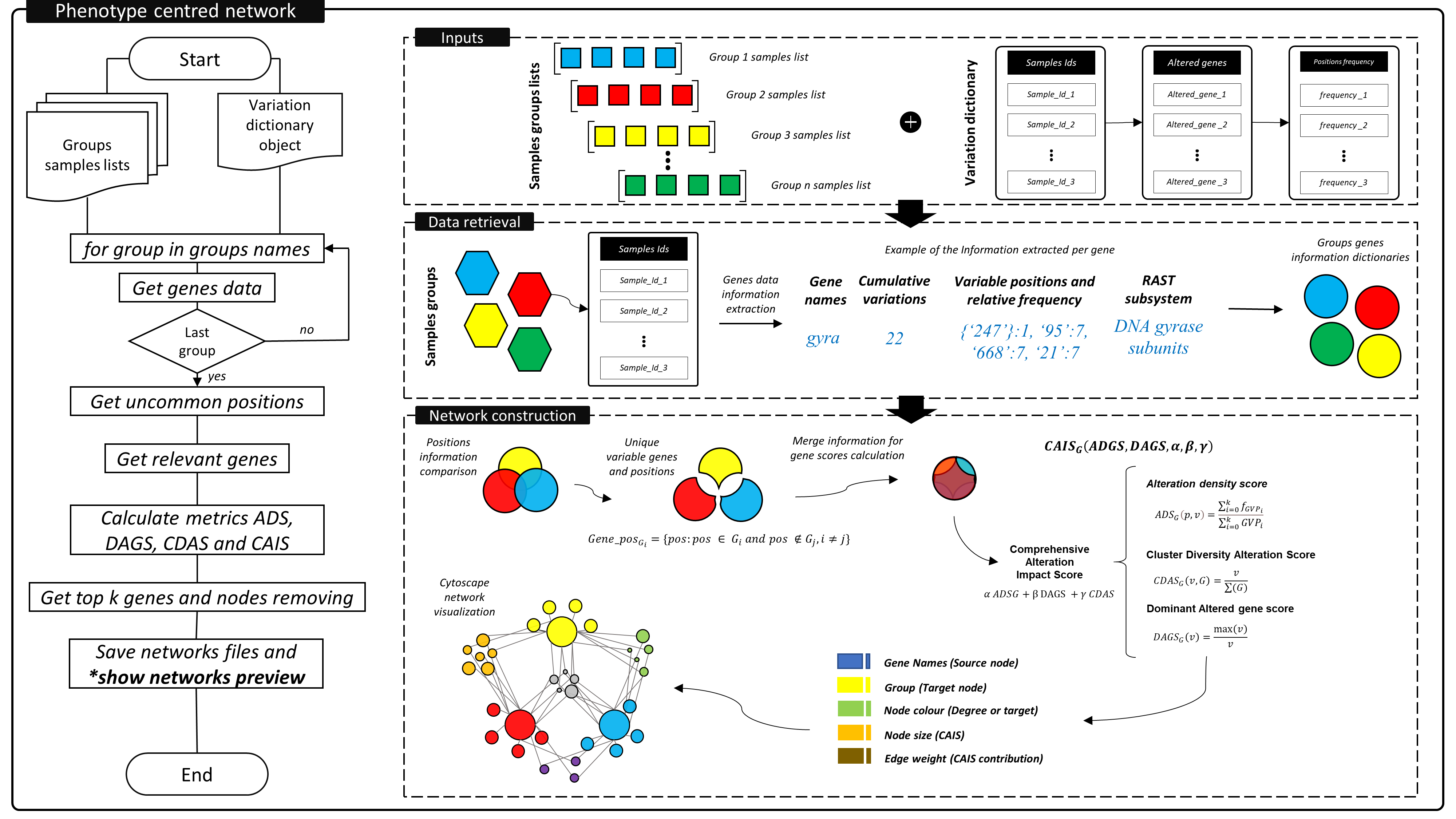

Supplement: btaf341_Supplementary_Data [file btaf341_supplementary_data.zip › Supplementary_Figure_4.TIF]

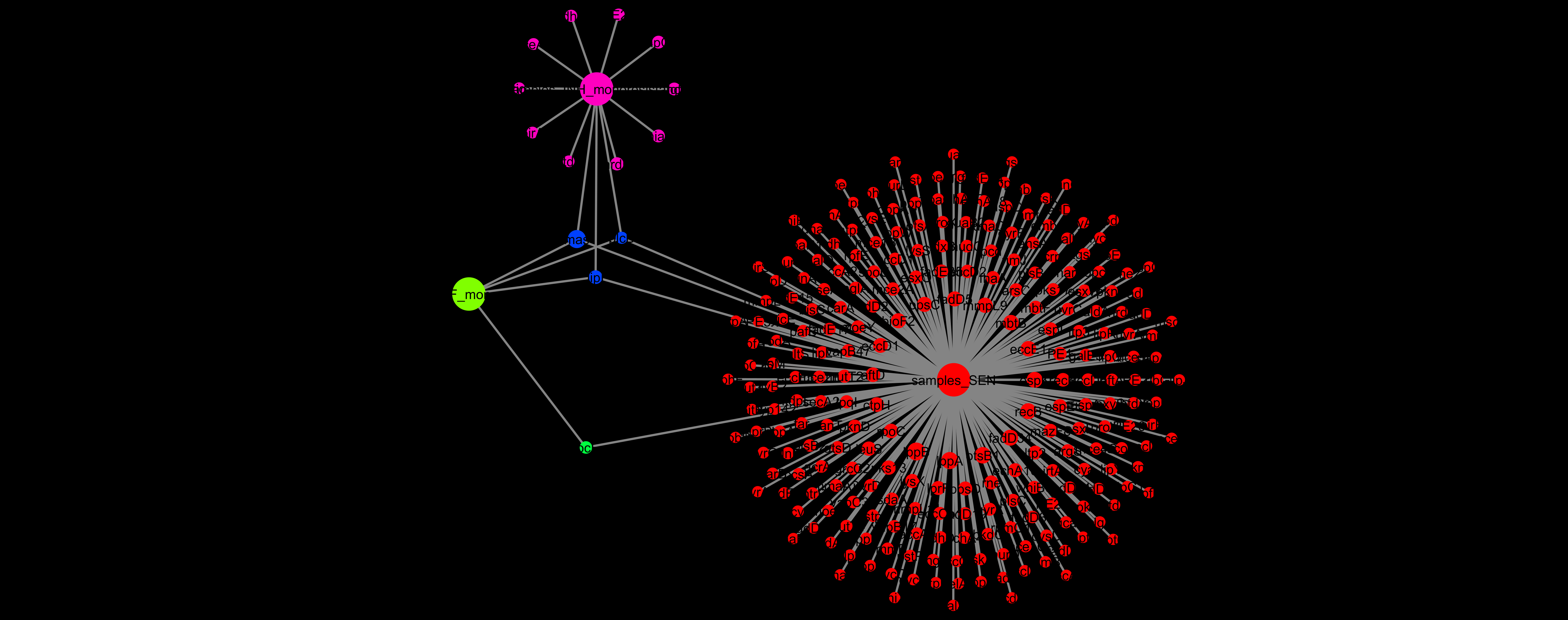

Supplement: btaf341_Supplementary_Data [file btaf341_supplementary_data.zip › Supplementary_figure_5_2.tiff]

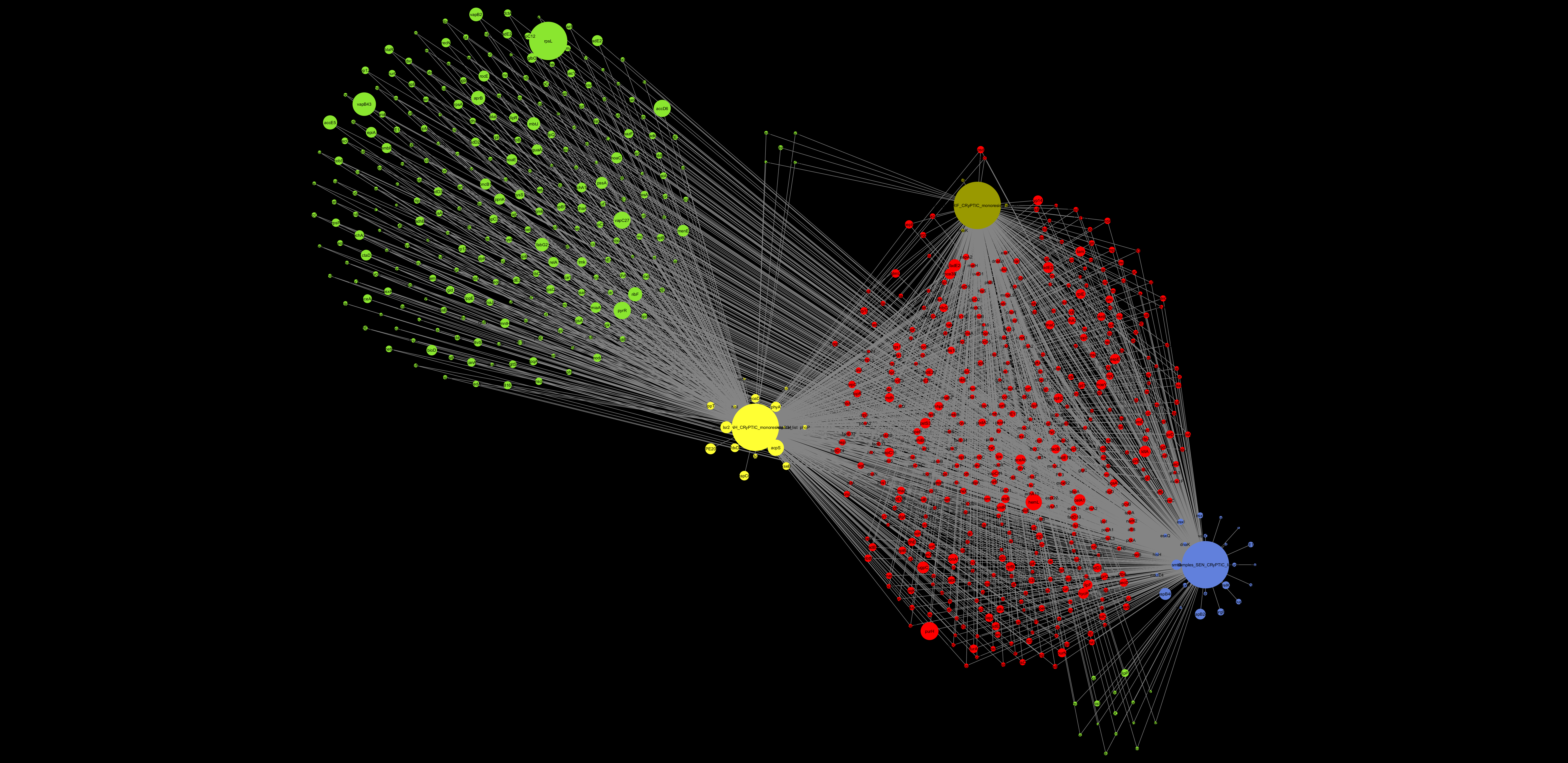

Supplement: btaf341_Supplementary_Data [file btaf341_supplementary_data.zip › Supplementary_Figure_6_2.tiff]
